# Role of the platelet-lymphocyte ratio as a prognostic indicator in patients with intracranial hemorrhage: A systematic review and meta-analysis

**DOI:** 10.1371/journal.pone.0311153

**Published:** 2025-02-10

**Authors:** Xiang Yuan, Sen Zhang, Jun Wan, Jingxian Yang, Yongjie Deng, Yuning Feng, Qingyu Bao, Xin Liu, Yihong Shen, Xian Chen, Jingyao Zeng, Yu Zhang

**Affiliations:** 1 Center for Evidence-based Medicine, Affiliated Hospital of Chengdu University, Chengdu, Sichuan, China; 2 Department of Critical Care Medicine, Affiliated Hospital of Chengdu University, Chengdu, Sichuan, China; 3 Department of Sports Training, Physical Culture Institute of Northeast Normal University, Changchun, Jilin, China; Sai Gosavi Specialty Clinic / Nano Hospitals Bangalore / Saraswati Specialty Clinic, INDIA

## Abstract

**Background:**

The prognostic value of platelet-lymphocyte ratio (PLR) in ischemic stroke had been investigated in previous studies. However, the results of studies on PLR in patients with intracranial hemorrhage (ICH) are inconsistent. We aimed to conduct a meta-analysis to determine the prognostic value of PLR in predicting functional outcome and mortality in patients with ICH.

**Methods:**

We searched the databases of PubMed, Embase, the Cochrane Library, and CNKI for relevant studies up to 10th June 2024. The Newcastle Ottawa Quality Assessment Scale (NOS) was applied to evaluate the quality of the included studies. We calculated the pooled odds ratios (OR) with 95% confidence intervals (CI) between PLR and both functional outcome (as measured by the modified Rankin Scale, mRS) as well as mortality. Poor functional outcomes were defined as mRS > 2.

**Results:**

A total of 6 studies with 2992 patients were included. The random effects meta-analysis demonstrated that elevated PLR exhibited an association with poor functional outcome in patients with ICH (OR = 1.69; 95% CI [1.39–2.07]; P<0.0001; I^2^ = 24%). Similarly, elevated PLR was associated with mortality in patients with ICH (OR = 1.65; 95% CI [1.12–2.43]; P = 0.01; I^2^ = 31%).

**Conclusion:**

This study suggested that elevated PLR was significantly associated with poor functional outcome (mRS>2) and increased mortality, indicating that elevated PLR could serve as a reliable a prognostic factor for unfavorable clinical outcomes in patients with ICH. It is advisable to conduct extensive prospective investigations across diverse ethnic backgrounds to verify the accuracy of this correlation prior to its utilization in clinical settings.

## Introduction

Intracranial hemorrhage (ICH) poses a significant mortality threat and frequently resulting in survivors experiencing varying degrees of residual disability [1, 2]. In 20–40% patients with ICH, hematoma growth could lead to early neurological deterioration. This contributes to a higher rate of disability and mortality for patients with ICH [3]. Hence, early identification of high-risk patients is of great significance for clinical treatment and family care [4]. The prognosis of patients with ICH is influenced by a complex interplay of various factors, including the size and location of the hemorrhage, patient comorbidities, and the underlying pathological mechanisms [5–7].

Increasing studies has proven that ICH initiates an inflammatory response. [8–10]. Systemic inflammatory markers play a crucial role in the diagnosis and prognostic assessment of intracranial hemorrhage. These markers include, but are not limited to, C-reactive protein, interleukin-6, tumor necrosis factor alpha, fibrinogen, and the platelet-lymphocyte ratio (PLR). Among these markers, the PLR is an affordable, available and composite biomarker for the inflammation of cerebrovascular disease. It combines the prognostic value of single platelet and lymphocyte counts in the field of ICH [11]. Previous studies have demonstrated a correlation between PLR and the severity as well as prognosis of various inflammation-linked conditions, including myocardial infarction, chronic autoimmune disorders, and peripheral ischemia [12–14].

The relationship between PLR and outcomes in patients with ICH is still controversial. Some studies found that elevated PLR levels are correlated with outcomes in patients with ICH [15–18]. However, some studies failed to find correlation between PLR and outcomes in patients with ICH [19, 20]. Therefore, we conducted this meta-analysis in order to determine the relationship between PLR and outcomes in patients with ICH.

## Methods

### Search strategy

The systematical search was performed in databases including PubMed, Embase, the Cochrane Library, and China National Knowledge Infrastructure (CNKI) without language limitation. The search encompassed the entire duration from the establishment of these databases to 10th, June 2024. Two reviewers conducted literature search and preliminary screening of the literature, independently. Key words used included a combination of terms including “Platelet to lymphocyte ratio”, “PLR”, “intracranial hemorrhage”. The search was further expanded by manually checking related references. When there was an inconsistency, it was resolved through discussion or determined by a third reviewer to ensure the stability of the results. The details of the search strategy are seen in S1 Table.

### Inclusion and exclusion criteria

The criteria for inclusion were listed as follows: (1) the patients were diagnosed with intracranial hemorrhage; (2) age ≥ 18 years old; (3) the measured outcome indicators include functional outcome, evaluated by the modified Rankin Scale (mRS), or mortality; (4) studies supplied sufficient information for calculating odds ratio (OR) and 95% confidence interval (CI). The exclusion criteria were as follows: (1) letters, case-reports, conference abstracts without original data; (2) reporting insufficient data for calculating an OR and 95% CI; (3) overlapping or duplicate data.

### Extraction of data

Two authors independently collected information from each included eligible study. The recorded information covering the first author, year of publication, study design, country of study, sample size, age, follow-up time, cutoff value of PLR, and the corresponding odds ratio (OR) and 95% CI values of mortality.

### Outcomes

In this analysis, the outcomes include functional outcome, evaluated by the modified Rankin Scale (mRS) where poor functional outcome was defined as mRS > 2, and mortality in patients with ICH.

### Quality assessment

The quality of each study was assessed in accordance with the Newcastle-Ottawa Scale (NOS), [21] which encompassed an evaluation of subject selection, comparability of groups, and clinical outcome. A total of nine items were extracted, and each item was awarded a score of 1. Two authors utilized the NOS to appraise the quality of the eligible studies. The total scores ranged from 0 to 9. A high-quality study was characterized as the one scoring≥7 points.

### Statistical analysis

The Review Manager (Version 5.3; Cochrane Collaboration) and STATA software were used for the statistics. OR and 95% CI were employed to assess the association of PLR with poor functional outcome (mRS>2) and mortality. Cochran’s Q test and Higgins I^2^ statistics were utilized to assess the heterogeneity of the combined data. Significant heterogeneity of data was defined as I^2^ > 50% and P < 0.1. A random-effects model was adopted in all of our studies. To address potential publication bias, we conducted quantitative analyses using Egger’s and Begg’s tests to comprehensively evaluate any potential biases stemming from small study effects. P < 0.05 was considered statistically significant for publication bias. We conducted sensitivity analyses for the meta-analysis on poor functional outcome (mRS>2) and mortality. In these analyses, individual studies were excluded one at a time, and the effect size was recalculated for the remaining studies within the meta-analysis software itself. Subgroup analyses were conducted based on the country of publication, NOS score, average age, the cut-off value of PLR, and sampling time.

## Results

### Search results

**Fig 1:**
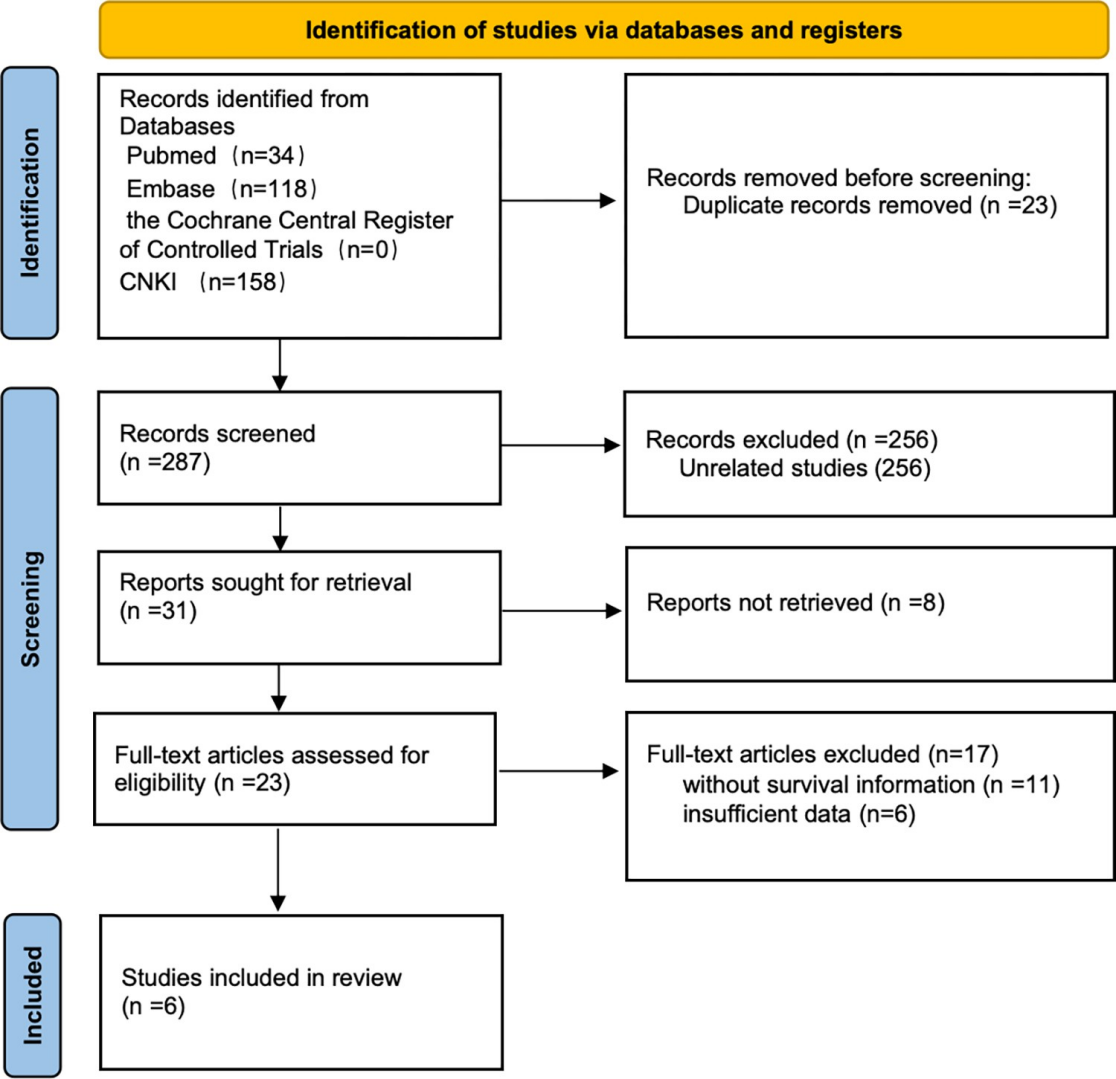
Flow diagram of the study selection process.

A total of 310 articles from the primary literature were found in the databases of PubMed, Embase, the Cochrane Library, and CNKI. Initially, 23 duplicates were identified and promptly eliminated. Following a rigorous screening process of titles and abstracts, a further 260 records were excluded as they did not meet the inclusion criteria. Of the remaining 31 articles, 8 were discarded due to the unavailability of their full-text versions. 11 were excluded because they lacked survival information, and another 6 were omitted due to insufficient data. Finally, six articles were chosen for inclusion in the meta-analysis (Fig 1).

### Eligible study characteristics

The characteristics of the six articles are summarized in Table 1. Most of these studies have been published since 2023. These studies included a total of 2,992 patients, and the number of patients in each study varied from 183 to 1,043. In terms of the methodological quality of the studies, the overall NOS scores ranged from 6 to 8 (S2 Table).

**Table 1:** Study characteristics of included studies for meta-analysis.

Author	Year	Country	Types of intracranial hemorrhage	Mean, age (SD)	Sample size	Male, N (%)	Design	Outcome measure	Optimal cut-off value	NOS score
Min Yuan [16]	2023	China	Intracerebral Hemorrhage	68.2 (15.1)	1043	570 (54.7)	Retrospective	3-month mortality	145.54	6
Yejin Kim [20]	2023	Korea	Intracerebral Hemorrhage	64.2 (15.6)	520	266 (62.0)	Retrospective	HE, 3-month poor functional outcome (mRS≥3), 1-month mortality	NR	7
Chuanyuan Tao [15]	2017	China	Aneurysmal Subarachnoid Hemorrhage	55.9 (11.9)	247	88 (35.6)	Prospective	DCI,3-month poor functional outcome (mRS≥3)	181.6	8
Weimin Zhang [19]	2018	China	Intracerebral Hemorrhage	52.4 (15.1)	183	117 (63.9)	Retrospective	6-months poor functional outcome (mRS≥3), GCS at hospital discharge	100	8
Seonong Yun [17]	2021	Korea	Aneurysmal Subarachnoid Hemorrhage	56.4 (13.2)	544	131 (33.4)	Retrospective	3-months poor functional outcome (mRS≥3)	130	8
Heling Chu [18]	2023	China	Intracerebral Hemorrhage	62.28 (13.3)	455	332 (73.0)	Retrospective	3-month poor functional outcome (mRS>3),1-month mortality	NR	7

SD: standard deviation; HE: hematoma expansion; DCI: delayed cerebral ischemia; mRS: modified Rankin Scale; GCS: Glasgow Coma Scale; NR: Not Reported

#### Functional outcome

**Fig 2:**
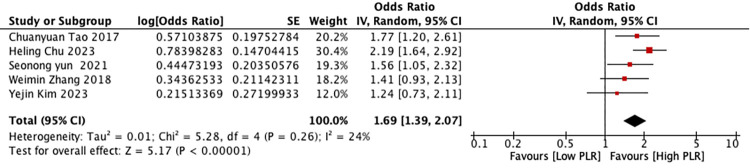
Forest plots for the association between platelet–lymphocyte ratio and poor functional outcome (mRS>2).

Five studies reported data on the association between PLR and poor functional outcome (mRS>2) of patients with ICH. The pooled analysis demonstrated that elevated PLR was associated with poor functional outcome (mRS>2). (OR = 1.69; 95% Cl [1.39–2.07]; P<0.0001; I^2^ = 24%; Fig 2).

### Mortality

**Fig 3:**
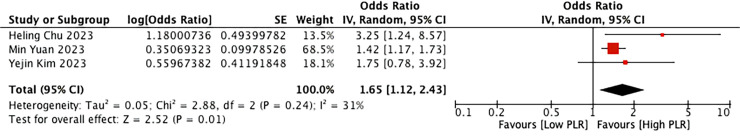
Forest plots for the association between platelet–lymphocyte ratio and mortality.

Three studies illustrated the association between PLR and overall mortality. The pooled analysis demonstrated that elevated PLR had an association with higher mortality (OR = 1.65;95% CI [1.12–2.43]; P = 0.01; I^2^ = 31%; Fig 3).

### Subgroup analysis

To detect potential heterogeneity, subgroup analyses were stratified based on the country of publication, NOS score, average age, the cut-off value of PLR, and sampling time (Table 2). The results of the subgroups revealed that none of the following factors contributed significantly to the source of heterogeneity: country of publication (China vs. Korea, P for interaction = 0.25), NOS score (NOS = 7 vs. NOS>7, P for interaction = 0.77), average age (average age≤60 vs. average age>60, P for interaction = 0.77), sample size (sample size≤450 vs. sample size>450, P for interaction = 0.75), the cut-off value of PLR (PLR≤130 vs. PLR>130, P for interaction = 0.48), and sampling time (retrospective vs. prospective, P for interaction = 0.76).

**Table 2:** Subgroup analysis of the association between platelet–lymphocyte ratio and poor functional outcome (mRS>2).

Factors	No. of studies	No. of patients	Random-effects model		Heterogeneity		Subgroup differences
			OR (95%)	P1 value	I^2^ (%)	P2 value	P3 value
Overall	5	1949	1.69 (1.39–2.07)	<0.00001	24	0.26	
Country							0.25
China	3	885	1.82 (1.42–2.35)	<0.00001	34	0.22	
Korea	2	1064	1.44 (1.04–1.98)	0.03	0	0.5	
NOS score							0.77
= 7	2	975	1.73(1.00–2.99)	0.07	70	0.26	
>7	3	974	1.58(1.26–1.99)	<0.0001	0	0.73	
Average age							0.77
≤60	3	974	1.58 (1.26–1.99)	<0.0001	0	0.73	
>60	2	975	1.73 (1.00–2.99)	0.05	70	0.07	
Sample size							0.75
≤450	2	430	1.59 (1.20–2.11)	0.001	0	0.43	
>450	3	1519	1.71 (1.23–2.37)	0.001	52	0.13	
Cutoff value							0.48
≤130	2	727	1.49 (1.11–1.98)	0.007	0	0.73	
>130	1	247	1.77 (1.26–1.99)	0.004	NA	NA	
Sampling time							0.76
Prospective	1	247	1.77 (1.26–1.99)	0.004	NA	NA	
Retrospective	4	1702	1.65 (1.27–2.14)	0.0002	43	0.15	

OR: odds ratio; Cl confidence interval; P1: P value for statistical based on Z test; P2_:_ P value for heterogeneity on Q test; P3: P value for interaction; NA: not applicable

### Sensitivity analysis

We removed 1 study each time to check the influence of the individual data set on the pooled ORs of poor functional outcome (mRS>2) (S3 Table) and mortality (S4 Table). The combined OR and its 95% CIs were not obviously affected. The result confirmed the robustness of the outcome of this study.

### Publication bias

The results of the Egger’s test indicate that the small-study effect did not demonstrate significant statistical significance in terms of mortality (P = 0.301), whereas it exhibited statistical significance in terms of poor functional outcome (mRS>2) (P = 0.01).

## Discussion

This meta-analysis assessed six studies involving 2,992 patients. The results of our meta-analysis indicated that an elevated PLR was significantly associated with poor functional outcome (mRS>2) and increased mortality.

To our knowledge, there has been no meta-analysis evaluating the association between PLR and outcomes in patients with ICH. Thus, this study represents the first meta-analysis to explore the prognostic significance of PLR in patients with ICH. Despite previous research suggesting its potential as an early prognostic indicator in major diseases, including stroke [22, 23], much of the existing research has notably concentrated on patients with ischemic stroke. For instance, a meta-analysis conducted by Divyansh et al. [24] identified a correlation between elevated PLR and poor ischemic stroke outcomes, including morbidity, mortality, and safety concerns. Our research has bridged this gap and highlighted the critical role of PLR in forecasting outcomes specifically in patients with ICH.

Current understanding of the cause for the association between elevated PLR in ICH patients and poor functional outcome is incomplete. However, several mechanisms may suggest a potential link. Firstly, platelet activation and the release of various inflammatory mediators are observed immediately after ICH, indicating a rapid systemic inflammatory response [25]. This response may not be merely coincidental, as an incremental increase in platelet activation and inflammation correlates with the severity of ICH and early brain injury. Inflammation in ICH could trigger blood-brain barrier disruption, neuronal cell death, synaptic damage, impairment of long-term potentiation, and white matter injury, all of which are significant contributors to a poor prognosis [26]. Secondly, inflammatory molecules and signaling pathways have been implicated in secondary brain injury following ICH. These include the activation of microglia and the infiltration of peripheral inflammatory cells, which are known to exacerbate brain injury [27]. The elevated PLR, serving as a proxy for an inflammatory milieu, can serve as an indicator of an ongoing inflammatory response, which significantly contributes to the severity and progression of ICH. Thirdly, PLR has emerged as a marker of systemic inflammation and has been associated with the development of extracerebral complications, including infections and cardiovascular events, which are frequently encountered among patients suffering from ICH [28]. These complications can significantly impact the overall prognosis and recovery of patients following ICH. Lastly, the prognostic value of PLR in ICH may stem from its reflection of platelet activity and the delicate balance between pro-inflammatory and anti-inflammatory processes. Elevated PLR could signify a heightened pro-thrombotic state and inflammation, both detrimental to the healing process and neurological recovery after ICH.

Systemic inflammatory response, a pivotal physiological reaction following ICH, encompasses the release of diverse inflammatory mediators such as C-reactive protein, interleukin-6, and tumor necrosis factor-α. These inflammatory markers not only mirror the body’s stress response to injury but also intimately correlate with the severity, disease progression, and patient prognosis of ICH. Existing research underscores that elevated levels of systemic inflammatory markers often portend a poorer prognosis for ICH patients. For instance, high CRP levels have been validated to be associated with increased rates of disability and mortality post-ICH. Similarly, the elevation of inflammatory cytokines like IL-6 and TNF-α is also recognized as an independent risk factor for adverse outcomes in ICH. These findings underscore the paramount importance of systemic inflammatory markers in the prognostic assessment of ICH patients. While the present study primarily focuses on the exploration of PLR as a prognostic indicator, it is imperative not to overlook the potential intricate interplay between PLR and systemic inflammatory markers. An increase in PLR may reflect the intensification of systemic inflammatory response, which, in turn, could further modulate the dynamics of PLR. This intricate relationship underscores the need for a comprehensive understanding of the interplay between these biomarkers in the context of ICH prognosis.

Our research revealed a significant correlation between an elevated PLR and both poor functional outcome (mRS > 2) and increased mortality among patients with ICH. While the current data does not identify a specific cut-off value for risk stratification, our findings suggest that PLR may serve as an accessible and cost-effective indicator for clinicians to consider when monitoring patients with ICH. This association prompts us to broaden our focus beyond the hemorrhage itself and to meticulously monitor patients’ platelet and lymphocyte levels. By identifying patients with elevated PLR levels, clinicians may be able to devise more targeted management strategies, potentially leading to more intensive monitoring, interventional measures, or tailored rehabilitation efforts.

When confronted with the coexistence of ICH and sepsis, a thorough analysis is required to accurately assess the inflammatory response and the prognostic significance of PLR from multiple perspectives. Despite both conditions involving inflammatory responses, their pathophysiological mechanisms differ significantly. ICH-induced inflammation is primarily localized to the brain tissue, potentially exacerbating brain injury, whereas sepsis is a systemic inflammatory response characterized by complex immune activation and cytokine storm. In this context, PLR, as a novel inflammatory marker, retains its utility in reflecting the degree of systemic inflammation. Although sepsis may elevate PLR, its changes can still indicate the inflammatory state of ICH patients.

To accurately assess the inflammatory response, we recommend integrating serum inflammatory markers (e.g., C-reactive protein and interleukin-6) with clinical manifestations and imaging changes for a comprehensive understanding of the patient’s condition. Additionally, the predictive effect of PLR may be influenced by factors such as patient age, gender, and underlying diseases, necessitating individualized analysis based on other clinical information when evaluating its value. Future studies can further explore the specificity and sensitivity of PLR in different inflammatory conditions to verify its potential application in clinical practice.

Nevertheless, several limitations of this study should be highlighted. Firstly, the most studies we included were retrospective in nature with selection bias, potentially impacting the reliability of our findings. Secondly, heterogeneity in studies was greater than expected due to potential variations in study populations, methodologies and definitions of outcomes that influence the overall findings, such diversity could significantly influence the aggregated results. Thirdly, each study had a different cut-off for PLR. It is possible that this could contribute to the heterogeneity. Furthermore, in relation to poor functional outcome (mRS>2), the Egger’s test indicates that the small-study effect has significant statistical significance. Lastly, the studies included in this analysis were exclusively conducted in China and Korea. This geographic limitation may raise concerns about the generalizability of our findings to other regions and populations. Future studies should aim to include a more diverse range of countries and populations to further validate our results.

## Conclusions

This study suggested that elevated PLR was significantly associated with poor functional outcome (mRS>2) and increased mortality, indicating that elevated PLR could serve as a reliable a prognostic factor for unfavorable clinical outcomes in patients with ICH. It is advisable to conduct extensive prospective investigations across diverse ethnic backgrounds to verify the accuracy of this correlation prior to its utilization in clinical settings.

## Supporting information

S1 Checklist PRISMA 2020 checklist. (DOCX)

S1 Table Search strategies. (DOCX)

S2 Table Newcastle–Ottawa scale score. (DOCX)

S3 Table Sensitivity analysis of meta-analysis between platelet–lymphocyte ratio and poor functional outcome. (DOCX)

S4 Table Sensitivity analysis of meta-analysis between platelet–lymphocyte ratio and mortality. (DOCX)

S5 Table List of excluded studies and included studies. (DOCX)
