# Supplementary material for: Role of the platelet-lymphocyte ratio as a prognostic indicator in patients with intracranial hemorrhage: A systematic review and meta-analysis
Source: PLoS One. 2025 Feb 10;20(2):e0311153. doi: 10.1371/journal.pone.0311153 (PMC11810451; doi:10.1371/journal.pone.0311153)
Supplement: S1 Table — (DOCX) [file pone.0311153.s002.docx]

**S2 Table. Search strategies.**

| PubMed | | |
| --- | --- | --- |
|  | "Blood Platelets"[Mesh] | 83416 |
|  | (Platelets) OR (Thrombocytes) OR (Blood Platelet) OR (Platelet) OR (Platelet, Blood) OR (Platelets, Blood) OR (Thrombocyte) | 329141 |
|  | (#1)OR(#2) | 329141 |
|  | "Lymphocytes"[Mesh] | 584287 |
|  | (Lymphoid Cells) OR (Cell, Lymphoid) OR (Cells, Lymphoid) OR (Lymphocyte) OR (Lymphoid Cell) | 878932 |
|  | (#4)OR(#5) | 878932 |
|  | (#3)AND(#6) | 21753 |
|  | "Hemorrhagic Stroke"[Mesh] | 690 |
|  | (Intracerebral Hemorrhage Stroke) OR (Intracerebral Hemorrhagic Stroke) OR (Subarachnoid Hemorrhagic Stroke) OR (Hemorrhage Stroke, Intracerebral) OR (Hemorrhagic Stroke, Intracerebral) OR (Hemorrhagic Stroke, Subarachnoid) OR (Hemorrhagic Strokes) OR (Intracerebral Hemorrhage Strokes) OR (Intracerebral Hemorrhagic Strokes) OR (Stroke, Hemorrhagic) OR (Stroke, Intracerebral Hemorrhage) OR (Stroke, Intracerebral Hemorrhagic) OR (Stroke, Subarachnoid Hemorrhagic) OR (Subarachnoid Hemorrhagic Strokes) | 23366 |
|  | (#8)OR(#9) | 23366 |
|  | (#7)AND(#10) | 34 |
| Cochrane | | |
|  | MeSH descriptor: [Lymphocytes] explode all trees | 7169 |
|  | ("Lymphocyte" OR " Cell, Lymphoid" OR " Cells, Lymphoid" OR " Lymphoid Cell" OR " Lymphoid Cells"):ti,ab,kw | 21171 |
|  | #1 OR #2 | 23968 |
|  | MeSH descriptor: [Blood Platelets] explode all trees | 2526 |
|  | ("Platelet" OR " Blood Platelet" OR " Thrombocytes" OR " Platelets" OR " Platelets, Blood" OR " Thrombocyte" OR " Platelet, Blood"):ti,ab,kw | 37974 |
|  | #4 OR #5 | 37974 |
|  | #3 AND #6 | 1399 |
|  | MeSH descriptor: [Hemorrhagic Stroke] explode all trees | 77 |
|  | ("Subarachnoid Hemorrhagic Strokes" OR " Subarachnoid Hemorrhagic Stroke" OR " Hemorrhagic Stroke, Subarachnoid" OR " Stroke, Subarachnoid Hemorrhagic" OR " Hemorrhage Stroke, Intracerebral" OR " Intracerebral Hemorrhage Strokes" OR " Intracerebral Hemorrhage Stroke" OR " Stroke, Intracerebral Hemorrhagic" OR " Intracerebral Hemorrhagic Stroke" OR " Intracerebral Hemorrhagic Strokes" OR " Stroke, Intracerebral Hemorrhage" OR " Hemorrhagic Stroke, Intracerebral" OR " Hemorrhagic Strokes" OR " Stroke, Hemorrhagic"):ti,ab,kw | 226 |
|  | #8 OR #9 | 299 |
|  | #7 AND #10 | 0 |
| EmBase | | |
|  | 'platelet lymphocyte ratio'/exp | 8867 |
|  | 'platelet lymphocyte ratio' OR 'platelet to lymphocyte ratio' OR 'platelet/lymphocyte ratio' OR 'plr (lymphocyte)' OR 'thrombocyte lymphocyte ratio' OR 'platelet lymphocyte ratio' | 9822 |
|  | #1 OR #2 | 9822 |
|  | 'brain hemorrhage'/exp | 193742 |
|  | 'brain hemorrhage' OR 'bleeding, corpus callosum' OR 'brain bleeding' OR 'brain haemorrhage' OR 'brain haemorrhage, traumatic' OR 'brain hemorrhage, traumatic' OR 'brain microhaemorrhage' OR 'brain microhemorrhage' OR 'brain stem haemorrhage, traumatic' OR 'brain stem hemorrhage, traumatic' OR 'cerebral haemorrhage' OR 'cerebral haemorrhage, traumatic' OR 'cerebral hemorrhage' OR 'cerebral hemorrhage, traumatic' OR 'cerebral microbleed' OR 'corpus callosum bleeding' OR 'corpus callosum haemorrhage' OR 'corpus callosum hemorrhage' OR 'encephalorrhagia' OR 'haemorrhage, brain' OR 'haemorrhage, intracranial' OR 'haemorrhagic apoplexy' OR 'haemorrhagic stroke' OR 'haemorrhagic stroke intracerebral bleeding' OR 'hematencephalon' OR 'hemorrhage, brain' OR 'hemorrhage, intracranial' OR 'hemorrhagic apoplexy' OR 'hemorrhagic stroke' OR 'hemorrhagic stroke intracerebral bleeding' OR 'hypertensive intracranial haemorrhage' OR 'hypertensive intracranial hemorrhage' OR 'intracerebral bleeding' OR 'intracerebral haemorrhage' OR 'intracerebral hemorrhage' OR 'intracortical haemorrhage' OR 'intracortical hemorrhage' OR 'intracranial bleeding' OR 'intracranial haemorrhage' OR 'intracranial haemorrhage, hypertensive' OR 'intracranial haemorrhage, traumatic' OR 'intracranial haemorrhages' OR 'intracranial hemorrhage' OR 'intracranial hemorrhage, hypertensive' OR 'intracranial hemorrhage, traumatic' OR 'intracranial hemorrhages' OR 'intraventricular haemorrhage' OR 'intraventricular hemorrhage' OR 'periventricular haemorrhage' OR 'periventricular hemorrhage' OR 'posterior fossa haemorrhage' OR 'posterior fossa hemorrhage' OR 'traumatic brain haemorrhage' OR 'traumatic brain hemorrhage' OR 'traumatic brain stem haemorrhage' OR 'traumatic brain stem hemorrhage' OR 'traumatic cerebral haemorrhage' OR 'traumatic cerebral hemorrhage' OR 'traumatic intracranial haemorrhage' OR 'traumatic intracranial hemorrhage' OR 'brain hemorrhage' | 163321 |
|  | #4 OR #5 | 208416 |
|  | #3 AND #6 | 118 |
| CNKI | | |
|  | platelet to lymphocyte ratio | 3617 |
|  | platelet-lymphocyte ratio | 3440 |
|  | platelet-to-lymphocyte ratios | 3054 |
|  | platelets to lymphocytes ratio | 59 |
|  | platelets-lymphocyte ratio | 1438 |
|  | platelet-to-lymphocyte count ratio | 24 |
|  | platelet to lymphocyte | 3182 |
|  | platelets and lymphocytes ratio | 5 |
|  | platelets to lymphocyte ratio | 2469 |
|  | platelet to lymphocyte ratio + platelet-lymphocyte ratio + platelet-to-lymphocyte ratios + platelets to lymphocytes ratio + platelets-lymphocyte ratio + platelet-to-lymphocyte count ratio + platelet to lymphocyte + platelets and lymphocytes ratio + platelets to lymphocyte ratio | 6949 |
|  | cerebral hemorrhage | 97806 |
|  | intracerebral hemorrhage | 107597 |
|  | intracranial hemorrhage | 33776 |
|  | hemorrhage | 690945 |
|  | acute cerebral hemorrhage | 8558 |
|  | intracerebral haemorrhage | 91047 |
|  | hypertensive cerebral hemorrhage | 28594 |
|  | apoplexy of brain | 88153 |
|  | cerebral hemorrhage + intracerebral hemorrhage + intracranial hemorrhage +  Hemorrhage + acute cerebral hemorrhage + intracerebral haemorrhage + hypertensive cerebral hemorrhage + apoplexy of brain | 899008 |
|  | (platelet to lymphocyte ratio + platelet-lymphocyte ratio + platelet-to-lymphocyte ratios + platelets to lymphocytes ratio + platelets-lymphocyte ratio + platelet-to-lymphocyte count ratio + platelet to lymphocyte + platelets and lymphocytes ratio + platelets to lymphocyte ratio) AND (cerebral hemorrhage + intracerebral hemorrhage + intracranial hemorrhage +  Hemorrhage + acute cerebral hemorrhage + intracerebral haemorrhage + hypertensive cerebral hemorrhage + apoplexy of brain) | 158 |
