# Supplementary material for: Role of the platelet-lymphocyte ratio as a prognostic indicator in patients with intracranial hemorrhage: A systematic review and meta-analysis
Source: PLoS One. 2025 Feb 10;20(2):e0311153. doi: 10.1371/journal.pone.0311153 (PMC11810451; doi:10.1371/journal.pone.0311153)
Supplement: S2 Table — (DOCX) [file pone.0311153.s003.docx]

**S3 Table. Newcastle–Ottawa scale Score.**

| Study | Selection | | | | Comparability | Outcome | | |
| --- | --- | --- | --- | --- | --- | --- | --- | --- |
|  | Representativeness of the exposed cohort | Selection of the non-exposed cohort | Ascertainment of exposure | Demonstration that outcome of interest was not present at start of study | Comparability of cohorts on the basis of the design or analysis | Assessment of outcome | Was follow-up long enough for outcomes to occur | Adequacy of follow up of cohorts |
| Min Yuan, 2023 | * |  | * | * | * | * | * |  |
| Yejin Kim, 2023 | * | * | * | * | * | * | * |  |
| Chuanyuan Tao, 2017 | * | * | * | * | * | * | * | * |
| Weimin Zhang, 2018 | * | * | * | * | * | * | * | * |
| Seonong Yun, 2021 | * | * | * | * | * | * | * | * |
| Heling Chu, 2023 | * | * | * | * | * | * | * |  |
