# Supplementary material for: Role of the platelet-lymphocyte ratio as a prognostic indicator in patients with intracranial hemorrhage: A systematic review and meta-analysis
Source: PLoS One. 2025 Feb 10;20(2):e0311153. doi: 10.1371/journal.pone.0311153 (PMC11810451; doi:10.1371/journal.pone.0311153)
Supplement: S3 Table — (DOCX) [file pone.0311153.s004.docx]

**S4 Table. Sensitivity analysis of meta-analysis between platelet–lymphocyte ratio and functional outcome.**

|  | **OR** | **95%CI** |
| --- | --- | --- |
| **All trials** | 1.69 | 1.39，2.07 |
| **Using a fixed-effect model** | 1.73 | 1.46，2.05 |
| **Excluding trials with Yejin Kim 2023** | 1.80 | 1.50，2.15 |
| **Excluding trials with Heling Chu 2023** | 1.52 | 1.23，1.88 |
| **Excluding trials with Seonong yun 2021** | 1.77 | 1.46，2.14 |
| **Excluding trials with Chuanyuan Tao 2017** | 1.72 | 1.42，2.08 |
| **Excluding trials with Weimin Zhang 2018** | 1.80 | 1.49，2.17 |
