# Supplementary material for: Role of the platelet-lymphocyte ratio as a prognostic indicator in patients with intracranial hemorrhage: A systematic review and meta-analysis
Source: PLoS One. 2025 Feb 10;20(2):e0311153. doi: 10.1371/journal.pone.0311153 (PMC11810451; doi:10.1371/journal.pone.0311153)
Supplement: S4 Table — (DOCX) [file pone.0311153.s005.docx]

**S5 Table. Sensitivity analysis of meta-analysis between platelet–lymphocyte ratio**

**and mortality.**

|  | **OR** | **95%CI** |
| --- | --- | --- |
| **All trials** | 1.65 | 1.12，2.43 |
| **Using a fixed-effect model** | 1.48 | 1.23，1.78 |
| **Excluding trials with Yejin Kim 2023** | 1.87 | 0.87，4.01 |
| **Excluding trials with Heling Chu 2023** | 1.44 | 1.19，1.74 |
| **Excluding trials with Min Yuan 2023** | 2.26 | 1.21，4.20 |
