# Supplementary material for: Role of the platelet-lymphocyte ratio as a prognostic indicator in patients with intracranial hemorrhage: A systematic review and meta-analysis
Source: PLoS One. 2025 Feb 10;20(2):e0311153. doi: 10.1371/journal.pone.0311153 (PMC11810451; doi:10.1371/journal.pone.0311153)
Supplement: S5 Table — (DOCX) [file pone.0311153.s006.docx]

**S6 Table. List of excluded studies and included studies.**

| **Unrelated studies（n=256）** |
| --- |
| 1. 安思怡. 妊娠期糖尿病患者孕中晚期TyG指数、PLR与新生儿出生体重的相关性分析. 硕士, 2023.  2. 蔡俊钦, 刘日阳, 卢锦丽, et al. 血小板淋巴细胞比值对儿童过敏性紫癜的预测价值研究. 当代临床医刊 2023; 36: 18-19.  3. 曹莹莹. 烟雾病患者外周血中炎症生物标志物的分析研究. 硕士, 2020.  4. 曾群. PLR及NLR对发热伴血小板减少综合征预后影响的研究. 硕士, 2019.  5. 陈彬. CysC、NLR、PLR与子痫前期严重程度的相关性研究. 硕士, 2023.  6. 陈杰翔. 炎症指标、血色素指标及血脂指标在膀胱尿路上皮癌中的表达水平及相关临床研究. 硕士, 2019.  7. 陈凯. 术前PLR、PNI对肝癌破裂出血患者部分肝切除术后肿瘤复发及死亡的预测价值. 硕士, 2020.  8. 陈小萍 and 陈泗林. 血小板和淋巴细胞比率对急性冠状动脉综合征患者住院和长期病死率的预测. 岭南心血管病杂志 2015; 21: 290-294.  9. 陈鑫. 术前血液炎症免疫营养指标在胃肠间质瘤预后中的应用价值研究. 硕士, 2021.  10. 崔彦. 外周血相关指标与丙型肝炎肝硬化并发上消化道出血的相关性分析. 硕士, 2021.  11. 崔彦 and 李成浩. 外周血相关指标与丙型肝炎肝硬化并发上消化道出血的相关性. 延边大学医学学报 2023; 46: 271-275. DOI: 10.16068/j.1000-1824.2023.04.007.  12. 邓彦玲. 妊娠期肝内胆汁淤积症患者妊娠结局的临床分析. 硕士, 2023.  13. 董新亚, 史永强, 米伟阳, et al. 新生儿颅内出血患儿外周血NLR水平的临床意义. 临床检验杂志(电子版) 2018; 7: 76-77.  14. 董艳丽 and 董淑娟. 替格瑞洛片联合阿司匹林片用于经皮冠状动脉介入术的急性ST段抬高型心肌梗死患者的临床研究. 中国临床药理学杂志 2019; 35: 614-616. DOI: 10.13699/j.cnki.1001-6821.2019.07.004.  15. 段宗奎. 结直肠癌患者术前炎症标志物与临床病理特征及术后并发症的相关性研究. 硕士, 2019.  16. 范倩倩. PLR、NLR联合血栓弹力图在妊娠合并血小板减少症患者母婴不良结局中的预测价值. 硕士, 2023.  17. 房鸿彬. 中性粒细胞-淋巴细胞比值等炎症标志物与高血压脑出血患者预后的相关性研究. 硕士, 2021.  18. 冯洁渊. 中性粒细胞/淋巴细胞比值和血小板/淋巴细胞比值与急性冠脉综合征的关系研究进展. 心血管病学进展 2020; 41: 1276-1280. DOI: 10.16806/j.cnki.issn.1004-3934.2020.12.013.  19. 付鹤鹏. 重症创伤患者临床指标及PLR联合MA值与死亡相关性分析. 硕士, 2018.  20. 高梦莹. 术前NLR、PLR对声门上型喉癌颈部淋巴结转移的预测价值. 硕士, 2022.  21. 耿新宇. 探究血常规相关指标联合PSA对前列腺癌的诊断价值及对前列腺穿刺的指导意义. 硕士, 2021.  22. 韩仟. 自发性脑出血患者死亡预测模型的开发和验证. 硕士, 2021.  23. 郝增光, 韩兆帅 and 葛慧敏. 血小板与淋巴细胞比值评估合并多支血管病变的急性心肌梗死患者预后的价值. 天津医科大学学报 2021; 27: 150-154.  24. 何海涛, 周泽, 魏志力, et al. 糖皮质激素对急性重症胰腺炎患者预后及炎症指标的影响. 中国药师 2023; 26: 435-441. DOI: 10.12173/j.issn.1008-049X.202311196.  25. 贺苗苗. FC、HBP、IL-6对克罗恩病活动及黏膜愈合的评估价值. 硕士, 2022.  26. 贺琪. 预测侧支循环最优HIR及其与继发性出血转化的相关性研究. 硕士, 2023.  27. 侯瑞华, 何尧利 and 温慧军. PLR和DFR结合WELLS评分对青年脑出血患者下肢深静脉血栓的预测价值. 中风与神经疾病杂志 2020; 37: 916-919. DOI: 10.19845/j.cnki.zfysjjbzz.2020.0484.  28. 胡锡慧. 287例儿童IgA血管炎临床特征及相关因素分析. 硕士, 2020.  29. 胡晓璐. 代谢和炎性因子预测妊娠期糖尿病的价值探讨. 硕士, 2022.  30. 黄凯涛. 过敏性紫癜消化道出血危险因素分析及血细胞参数价值. 硕士, 2022.  31. 蒋甘孺, 金彬彬, 张明珠, et al. 外周血中性粒细胞与淋巴细胞比值在维持性血液透析合并脑出血患者预后中的预测价值. 医学综述 2020; 26: 4127-4131+4136.  32. 蒋骞. 动脉瘤性蛛网膜下腔出血后早期脑损伤与外周炎症的相关性研究. 硕士, 2020.  33. 金彬彬, 刘亚 and 李胜开. 维持性血液透析合并脑出血患者血肿扩大的相关因素分析. 实用医学杂志 2020; 36: 1238-1242.  34. 荆玲 and 张馨. 卒中后出血转化与炎症反应相关性研究. 北华大学学报(自然科学版) 2024; 25: 82-86.  35. 李婵, 梁志海 and 唐国都. 重症急性胰腺炎继发持续性炎症-免疫抑制-分解代谢综合征的影响因素及预测模型构建. 临床肝胆病杂志 2023; 39: 1382-1390.  36. 李静兰. 血清D-DT联合肺部超声评分对早产儿支气管肺发育不良症的早期预测研究. 硕士, 2022.  37. 李坷. 机械取栓治疗后循环脑梗死预后的影响因素. 现代诊断与治疗 2021; 32: 2798-2800.  38. 李奇芳, 罗玲玲 and 黄淑华. 血小板/淋巴细胞和中性粒细胞/淋巴细胞比值与异常子宫出血的关系研究. 江西医药 2020; 55: 1850-1852.  39. 李强. 慢性肾衰竭合并脑出血的预后影响因素分析. 硕士, 2018.  40. 李瑞 and 胡蓉. 基于白细胞衍生性指标建立肝硬化失代偿期患者自发性细菌性腹膜炎短期预后风险预测模型的价值研究. 临床内科杂志 2023; 40: 34-37.  41. 李屾. PLR、LMR、D-dimer与颅内静脉窦血栓患者预后的关联研究. 硕士, 2019.  42. 李屾, 刘凯, 高远, et al. 相关炎性因子对静脉窦血栓患者预后的预测价值研究. 中国实用神经疾病杂志 2018; 21: 2338-2344.  43. 李世安, 李爱民, 陈军, et al. 中性粒细胞/淋巴细胞及血小板/淋巴细胞比值对脑损伤后进展性出血的预测价值. 临床神经外科杂志 2018; 15: 147-151.  44. 李天泽, 曲政, 吕博, et al. 动脉瘤性蛛网膜下腔出血急性期中性粒细胞/淋巴细胞比值、血糖/血钾比值与神经功能缺损程度的相关性及预测价值. 中国当代医药 2023; 30: 13-18.  45. 李雨璠. 血脂及外周血炎症指标与自发性脑出血预后关系的初步研究. 硕士, 2023.  46. 梁永慧. 白细胞介素17A对脓毒症小鼠多器官功能及预后影响的研究. 硕士, 2023.  47. 刘昊旻, 何云, 李小林, et al. 血常规预测过敏性紫癜患儿消化道出血指标的价值. 西南医科大学学报 2022; 45: 328-331.  48. 刘冉, 王宗方, 杨洋, et al. 冠状动脉内注射重组人尿激酶原治疗急性ST段抬高型心肌梗死的疗效. 西北药学杂志 2023; 38: 174-178.  49. 刘思晖. 术前外周血中性粒细胞与淋巴细胞比值和血小板与淋巴细胞比值评估肝癌预后的价值. 硕士, 2018.  50. 龙强友, 漆新伟 and 王德全. 高血压性脑出血外周血炎症标志物与术后30天病死率的关系. 中国临床神经外科杂志 2017; 22: 302-304. DOI: 10.13798/j.issn.1009-153X.2017.05.006.  51. 陆俊福. 血小板计数对失血性休克急性肾损伤及28天病死率影响的回顾性队列研究. 硕士, 2018.  52. 陆小芳, 周晓荣, 钱东林, et al. 吉西他滨注射液联合顺铂注射液治疗中晚期非小细胞肺癌患者的临床研究. 中国临床药理学杂志 2021; 37: 805-808. DOI: 10.13699/j.cnki.1001-6821.2021.07.004.  53. 吕麦扣, 侯瑞华 and 温慧军. 急性青年脑梗死血小板及淋巴细胞比值与出血转化的研究. 中风与神经疾病杂志 2021; 38: 711-713. DOI: 10.19845/j.cnki.zfysjjbzz.2021.0188.  54. 吕文蝶. NLR、PLR、MPV和急性缺血性脑卒中静脉溶栓后出血转化的相关性分析. 硕士, 2023.  55. 罗冬, 李国梁 and 王军文. 系统性免疫炎症指数对急性前循环大血管闭塞机械取栓首次通过效应的预测价值. 中国脑血管病杂志 2023; 20: 10-19.  56. 马聪聪. 宫颈癌患者术后总生存期相关影响因素和预测模型的建立. 硕士, 2018.  57. 马庆宇, 张景展, 梁俊琴, et al. 儿童及成人腹型过敏性紫癜临床特征分析. 新疆医学 2021; 51: 258-260+264.  58. 马昕. 胃肠胰神经内分泌肿瘤炎症相关因子的表达及其预后分析. 硕士, 2022.  59. 米娟 and 孙建芳. 淋巴细胞计数、NLR、PLR诊断不同类型胎膜早破价值. 中国计划生育学杂志 2021; 29: 1995-1999.  60. 倪晓鸽, 朱琳娜, 房玉珠, et al. 经阴道三维超声联合CA125、CA199、NLR及PLR检测对绝经后子宫内膜癌的诊断效能. 现代生物医学进展 2023; 23: 1771-1775. DOI: 10.13241/j.cnki.pmb.2023.09.033.  61. 庞琪, 杨合慧, 曾怀文, et al. 血液指标在自发性脑出血中的临床意义. 江苏医药 2024; 50: 245-249. DOI: 10.19460/j.cnki.0253-3685.2024.03.007.  62. 彭忠. 肝癌合并急性上消化道出血的治疗策略及预后评估. 硕士, 2018.  63. 彭舟丽, 淮瑞敏 and 张建霞. 小剂量阿司匹林预防高危孕妇子痫前期的价值分析. 中国实用医药 2024; 19: 120-122. DOI: 10.14163/j.cnki.11-5547/r.2024.07.030.  64. 祁孟丽, 陶雅非, 任东升, et al. 尿毒症行维持性透析并发脑出血患者预后的影响因素及风险模型构建. 中华实用诊断与治疗杂志 2020; 34: 44-47. DOI: 10.13507/j.issn.1674-3474.2020.01.012.  65. 钱宣辰, 李庆节 and 聂耳. PLR(血小板/淋巴细胞比值)、嗜酸性粒细胞绝对值计数预测急性脑梗死患者溶栓后出血转化的价值. 青岛医药卫生 2023; 55: 170-174.  66. 邵琳, 朱言芳, 赵国, et al. 外周血血小板/淋巴细胞比值、纤维蛋白原/白蛋白比值与缺血性卒中静脉溶栓后出血转化的相关性. 安徽医药 2023; 27: 1637-1641.  67. 申慧鑫, 孙蔚, 武霄, et al. 炎性反应相关指标对急性缺血性卒中患者血管内治疗临床预后的影响. 中国脑血管病杂志 2023; 20: 382-391.  68. 石琼娅. 老年自发性脑出血患者深静脉血栓形成的危险因素分析. 硕士, 2023.  69. 石琼娅, 周立宇, 张娇, et al. 预测老年自发性脑出血患者深静脉血栓——基于MIMIC-Ⅳ数据库回顾性分析. 临床神经外科杂志 2023; 20: 327-331.  70. 史汉童, 郭彦谷, 侯晓翔, et al. 血常规检查中各项参数对急性脑出血患者脑损伤程度及死亡风险预测价值. 临床军医杂志 2024; 52: 333-337. DOI: 10.16680/j.1671-3826.2024.04.02.  71. 宋佳忆, 肖璇, 陈婷, et al. 中性粒细胞与淋巴细胞比值对糖尿病性玻璃体积血的预测价值. 武汉大学学报(医学版) 2024; 45: 95-98+126. DOI: 10.14188/j.1671-8852.2022.0224.  72. 宋健 and 许铁. 中性粒细胞/淋巴细胞比值对急性缺血性脑卒中溶栓治疗预后的预测价值. In: 2018年浙江、江苏两省急诊医学学术年会 中国浙江嘉兴, 2018, p.2.  73. 宋伟. 血清eNOS联合肺部超声对早产儿支气管肺发育不良的早期预测研究. 硕士, 2021.  74. 苏亮. Fractalkine在心梗后PCI患者缺血再灌注损伤中的作用.  75. 苏鹏山. 急性脑梗死患者脑微出血相关因素及其与短期预后关系的分析. 硕士, 2023.  76. 孙杨. Glasgow预后评分及外周血炎症指标在脑出血患者中的临床意义. 硕士, 2018.  77. 汤国艳. mGPS评分和外周血炎症指标对脑出血患者短期预后的临床研究. 硕士, 2019.  78. 唐玥, 田雅丽, 颜伟, et al. 利伐沙班联合导管溶栓在老年颅内出血患者急性上肢深静脉血栓形成的血小板∕淋巴细胞比率及预后影响. 中华保健医学杂志 2024; 26: 22-25.  79. 汪彪, 卓柳安, 陈宇, et al. 高血压脑出血患者单核细胞/淋巴细胞比率及其临床意义. 广西医学 2018; 40: 1315-1317.  80. 王丹, 王新志, 陈婧, et al. 早期免疫营养干预对高血压脑出血患者免疫功能影响. 华南预防医学 2019; 45: 540-543.  81. 王晶晶, 赵新斌, 郭笑颜, et al. 基于动脉自旋标记的动脉瘤性蛛网膜下腔出血短期预后模型构建及评价:一项前瞻性研究. 分子影像学杂志 2024; 47: 19-24.  82. 王璐. 基于机器学习整合多维组学数据指导食管癌精准放疗的应用基础研究. 博士, 2021.  83. 王培研. FAR、PLR与特发性膜性肾病血栓栓塞事件的相关性研究. 硕士, 2021.  84. 王倩. 血小板和淋巴细胞比率与大动脉炎疾病活动性的相关研究. 硕士, 2017.  85. 王琼琼. 丁苯酞与尤瑞克林在大动脉粥样硬化性脑卒中患者治疗中的效果研究. 中国实用医药 2024; 19: 1-5. DOI: 10.14163/j.cnki.11-5547/r.2024.02.001.  86. 王书华. 肾癌的临床病例特点及预后相关因素的研究. 硕士, 2016.  87. 王淑玲. HSIL患者中存在隐匿性宫颈癌的影响因素分析及其列线图模型构建. 硕士, 2023.  88. 王玮. 急性心肌梗死后微血管阻塞的影响因素及其对左室重构的预测价值. 硕士, 2021.  89. 王小花, 林坚, 邢东文, et al. 血小板-淋巴细胞比值对儿童过敏性紫癜胃肠道出血的预测价值分析. 中国中西医结合皮肤性病学杂志 2023; 22: 163-164.  90. 王一茜. 儿童IgA血管炎294例临床特征回顾性研究. 硕士, 2022.  91. 吴辉. 替格瑞洛和氯吡格雷在PPCI治疗急性冠脉综合征的对比研究. 博士, 2022.  92. 吴为敏. 小肝癌及微小肝癌根治性切除术后肝内复发的临床病理因素分析. 硕士, 2019.  93. 武宁, 李健, 贾帅, et al. 依达拉奉右莰醇联合替罗非班治疗大动脉粥样硬化型进展性缺血性卒中的效果研究. 实用心脑肺血管病杂志 2022; 30: 96-101.  94. 闫海燕, 丁延魁, 郏红静, et al. 院前肝素化联合一包药与急性ST段抬高型心肌梗死患者血管再通的相关性. 临床荟萃 2023; 38: 232-236.  95. 杨俊囡. 血炎症标志物在间质性肺病相关肺动脉高压中的临床意义. 硕士, 2023.  96. 余静. NLR、MLR、PLR与炎症性肠病活动性的相关性分析. 硕士, 2021.  97. 余婷. 阿加曲班对进展性脑梗死患者的安全性及临床预后的影响. 硕士, 2022.  98. 袁虎方. 大株红景天对进展期胃癌患者XELOX方案新辅助化疗疗效及心脏保护作用的研究.  99. 张慧. 血小板参数对儿童过敏性紫癜及其胃肠道出血的预测价值. 硕士, 2018.  100. 张慧 and 南虎松. 血小板参数诊断儿童过敏性紫癜的临床意义. 吉林医学 2018; 39: 1836-1840.  101. 张科明, 胡行前, 吴益斌, et al. 术前PLR和NLR预测重度胆囊炎的效用. 南京医科大学学报(自然科学版) 2022; 42: 719-723.  102. 张鹏, 李育平, 王晓东, et al. 结合炎症反应指数的Nomogram模型对aSAH患者预后预测的价值. 临床神经外科杂志 2020; 17: 216-220.  103. 张盛楠. 脑微出血危险因素及其与认知功能相关性研究. 硕士, 2020.  104. 张文亮, 刘叶, 刘亮, et al. 脑出血病人合并卒中相关性肺炎不良预后的列线图预测模型构建. 中西医结合心脑血管病杂志 2023; 21: 739-744.  105. 张希萌. 中性粒细胞/淋巴细胞比值及血小板/淋巴细胞比值与伴系统损害儿童过敏性紫癜的相关性分析. 硕士, 2020.  106. 张希萌, 崔丽霞, 彭戈, et al. 中性粒细胞/淋巴细胞比值及血小板/淋巴细胞比值与伴系统损害儿童过敏性紫癜的相关性分析. 中国中西医结合皮肤性病学杂志 2022; 21: 132-136.  107. 张晓雪. 儿童腺样体肥大与外周血炎性指标及细菌学的相关性研究. 硕士, 2022.  108. 张亚. 急性轻中度脑梗死患者早期认知障碍的影响因素及其照料者负担的相关性研究. 硕士, 2023.  109. 张月战. 血循环miR-29b和miR-424与急性脑血管病的分类及短期预后的相关性研究. 博士, 2018.  110. 赵婷婷. 基于炎症标志物、眼动跟踪和~(18)F-SynVesT-1 PET探索CSVD的认知障碍. 博士, 2023.  111. 周岳 and 李晓辉. 胃癌患者肿瘤血液免疫指标与术后并发症的关系分析. 检验医学与临床 2022; 19: 2309-2313.  112. 朱飞飞. 多种炎症标志物检测对消化道出血的诊断价值. 医学理论与实践 2023; 36: 123-125. DOI: 10.19381/j.issn.1001-7585.2023.01.049.  113. 朱荣坤. LDH、PLR、NLR、SII及D-二聚体、RI对子宫肉瘤的联合诊断价值分析. 硕士, 2023.  114. 祖雪. 增生性糖尿病性视网膜病变玻璃体切割术后发生新生血管性青光眼的风险因素. 硕士, 2022.  115. 左丹丹. 胎龄＜32周早产儿BPD危险因素分析及NLR、PLR、SII对其临床应用价值. 硕士, 2022.  116. Ahn HJ, Kang J, Lee SR, et al. Neutrophil-to-lymphocyte ratio as a predictor of in-hospital complications and overall mortality in Takotsubo syndrome preceded by physical triggers. BMC Cardiovascular Disorders 2023; 23. Article. DOI: 10.1186/s12872-023-03078-1.  117. Akif TM, Yasir PM, Ezgi Y, et al. Systemic Inflammation Indices in Patients With Acute Ischemic Stroke Treated With Intravenous Tissue Plasminogen Activator: Clinical Yield and Utility. Angiology 2020; 72: 3319720969997-3319720969997.  118. Amaral LM, Cunningham MW, Jr., Cornelius DC, et al. Preeclampsia: long-term consequences for vascular health. Vasc Health Risk Manag 2015; 11: 403-415. 20150715. DOI: 10.2147/vhrm.S64798.  119. Bateman RM, Sharpe MD, Jagger JE, et al. 36th International Symposium on Intensive Care and Emergency Medicine : Brussels, Belgium. 15-18 March 2016. Crit Care 2016; 20: 94. 20160420. DOI: 10.1186/s13054-016-1208-6.  120. Bengzon DJD, Carmen P, Michael B, et al. Inflammatory Biomarkers and Intracranial Hemorrhage after Endovascular Thrombectomy. The Canadian journal of neurological sciences Le journal canadien des sciences neurologiques 2021; 49: 1-7.  121. Bilge H and Başol O. The effect of platelet-albumin ratio on mortality and morbidity in peptic ulcer perforation. Medicine (United States) 2022; 101: E29582. Article. DOI: 10.1097/MD.0000000000029582.  122. Cai C, Yan C, Chen S, et al. Development and Validation of a Prediction Model for 30-Day Mortality and Functional Outcome in Patients with Primary Brainstem Hemorrhage. Cerebrovascular Diseases 2024; 53: 79-87. Article. DOI: 10.1159/000530348.  123. Cai Z, Zhao K, Li Y, et al. Early Enteral Nutrition Can Reduce Incidence of Postoperative Hydrocephalus in Patients with Severe Hypertensive Intracerebral Hemorrhage. Medical Science Monitor 2022; 28. Article. DOI: 10.12659/MSM.935850.  124. Cakir U, Tayman C, Tugcu AU, et al. Role of Systemic Inflammatory Indices in the Prediction of Moderate to Severe Bronchopulmonary Dysplasia in Preterm Infants. Archivos de Bronconeumologia 2023; 59: 216-222. Article. DOI: 10.1016/j.arbres.2023.01.003.  125. Celkan TT. What does a hemogram say to us? Turkish Archives of Pediatrics 2020; 55: 103-116. Review. DOI: 10.14744/TurkPediatriArs.2019.76301.  126. Çevikkalp E and Taşkapılıoğlu MÖ. Predictive Values of Hematological Parameters for Determining Imminent Brain Death: A Retrospective Study. Medicina (Kaunas, Lithuania) 2023; 59. Article. DOI: 10.3390/medicina59020417.  127. Chen L, Xia S, Zuo Y, et al. Systemic immune inflammation index and peripheral blood carbon dioxide concentration at admission predict poor prognosis in patients with severe traumatic brain injury. Frontiers in Immunology 2023; 13. Article. DOI: 10.3389/fimmu.2022.1034916.  128. Chen L and Zhang Q. Increased Mean Platelet Volume is Associated with Poor Outcome in Patients with Aneurysmal Subarachnoid Hemorrhage. World Neurosurgery 2020; 137: e118-e125. Article. DOI: 10.1016/j.wneu.2020.01.068.  129. Chen W, Huang Y, Chong CM, et al. Editorial: Post-stroke complications: mechanisms, diagnosis, and therapies. Frontiers in Neurology 2023; 14. Editorial. DOI: 10.3389/fneur.2023.1292562.  130. Chen XY, Chen Y, Lin N, et al. A Nomogram for Predicting the Need of Postoperative Tracheostomy in Patients With Aneurysmal Subarachnoid Hemorrhage. Frontiers in Neurology 2021; 12. Article. DOI: 10.3389/fneur.2021.711468.  131. Chen Y, Liu J, Li Y, et al. The Independent Value of Neutrophil to Lymphocyte Ratio in Gouty Arthritis: A Narrative Review. Journal of Inflammation Research 2023; 16: 4593-4601. Review. DOI: 10.2147/JIR.S430831.  132. Chen Y, Tian J, Chi B, et al. Factors associated with the development of coagulopathy after open traumatic brain injury. Journal of Clinical Medicine 2022; 11. Article. DOI: 10.3390/jcm11010185.  133. Chen Z, He Y, Su Y, et al. Association of inflammatory and platelet volume markers with clinical outcome in patients with anterior circulation ischaemic stroke after endovascular thrombectomy. Neurol Res 2021; 43: 503-510. 20210105. DOI: 10.1080/01616412.2020.1870359.  134. Chuanyuan T, Jiajing W, Xin H, et al. Clinical Value of Neutrophil to Lymphocyte and Platelet to Lymphocyte Ratio After Aneurysmal Subarachnoid Hemorrhage. Neurocritical care 2017; 26: 393-401.  135. Cui X, Liu Q, Xia R, et al. Injury-Admission Time is an Independent Risk Factor for Deep Vein Thrombosis in Older Patients with Osteoporotic Hip Fracture. Medical Science Monitor 2024; 30. Article. DOI: 10.12659/MSM.943587.  136. Davies MG and Hart JP. Current status of ECMO for massive pulmonary embolism. Frontiers in Cardiovascular Medicine 2023; 10. Short Survey. DOI: 10.3389/fcvm.2023.1298686.  137. Defort P, Retkowska-Tomaszewska N, Kot M, et al. Inflammatory Predictors of Prognosis in Patients with Traumatic Cerebral Haemorrhage: Retrospective Study. Journal of Clinical Medicine 2022; 11. Article. DOI: 10.3390/jcm11030705.  138. Dettori P, Paliogiannis P, Pascale RM, et al. Blood cell count indexes of systemic inflammation in carotid artery disease: Current evidence and future perspectives. Current Pharmaceutical Design 2021; 27: 2170-2179. Review. DOI: 10.2174/1381612826666201222155630.  139. Diestro J, Farinas CP, Balas M, et al. P-003 Association between baseline serology and symptomatic intracranial hemorrhage after endovascular thrombectomy for acute ischemic stroke. Journal of NeuroInterventional Surgery 2021; 13: A26-A27.  140. Diestro J, Parra-Farinas C, Balas M, et al. Association between baseline serology and symptomatic intracranial hemorrhage after endovascular thrombectomy for acute ischemic stroke. Journal of NeuroInterventional Surgery 2021; 13: A26-A27. Conference Abstract. DOI: 10.1136/neurintsurg-2021-SNIS.39.  141. Diker S, Gelener P, Eker A, et al. Association between cerebral microbleeds and inflammatory biomarkers in patients with ischemic stroke. Egyptian Journal of Neurology, Psychiatry and Neurosurgery 2022; 58. Article. DOI: 10.1186/s41983-022-00478-6.  142. Dourmashkin LH, Lyons B, Hess RS, et al. Evaluation of the neutrophil-to-lymphocyte and platelet-to-lymphocyte ratios in critically ill dogs. Journal of Veterinary Emergency and Critical Care 2023; 33: 52-58. Article. DOI: 10.1111/vec.13269.  143. Duan H, Yang F, Peng Y, et al. Correlation of platelet parameter and platelet-lymphocyte ratio with severity and prognosis in patients with traumatic brain injury. International Journal of Clinical and Experimental Medicine 2020; 13: 8909-8914. Article.  144. Feng L, Hongyun Z, Yingkun H, et al. Relationship between first pass effect during mechanical thrombectomy and neutrophil to lymphocyte ratio in acute anterior circulation large vessel occlusive stroke. Chinese Journal of Neuromedicine 2022; 21: 132-138. Article. DOI: 10.3760/cma.j.cn115354-20211213-00813.  145. Feng X, Ye G, Cao R, et al. Identification of Predictors for Hemorrhagic Transformation in Patients with Acute Ischemic Stroke After Endovascular Therapy Using the Decision Tree Model. Clin Interv Aging 2020; 15: 1611-1624. 20200908. DOI: 10.2147/cia.S257931.  146. Fonseca S, Costa F, Seabra M, et al. Systemic inflammation status at admission affects the outcome of intracerebral hemorrhage by increasing perihematomal edema but not the hematoma growth. Acta Neurologica Belgica 2021; 121: 649-659. Article. DOI: 10.1007/s13760-019-01269-2.  147. Francoeur CL, Najjar A, Frédéric R, et al. Leucocyte profiles as simple immunomonitoring tools and their association with outcome in subarachnoid hemorrhage: A retrospective cohort study. Intensive Care Medicine Experimental 2018; 6. Conference Abstract. DOI: 10.1186/s40635-018-0201-6.  148. Fu S, Liu H, Wang G, et al. Incidence, risk factors, and clinical outcomes of acute brain swelling associated with traumatic acute subdural hematoma: a retrospective study utilizing novel diagnostic criteria. Therapeutic Advances in Neurological Disorders 2024; 17. Article. DOI: 10.1177/17562864241242944.  149. Garagoli F, Fiorini N, Pérez MN, et al. neutrophil-to-lymphocyte ratio and platelet-to-lymphocyte ratio predict in-hospital mortality in symptomatic but unruptured abdominal aortic aneurysm patients. International Angiology 2022; 41: 188-195. Article. DOI: 10.23736/S0392-9590.22.04754-X.  150. Guan J, Wang Q and Zhao Q. Lymphocyte to Monocyte Ratio is Independently Associated with Futile Recanalization in Acute Ischemic Stroke After Endovascular Therapy. Neuropsychiatric Disease and Treatment 2023; 19: 2585-2596. Article. DOI: 10.2147/NDT.S434225.  151. Guo P and Zou W. Neutrophil-to-lymphocyte ratio, white blood cell, and C-reactive protein predicts poor outcome and increased mortality in intracerebral hemorrhage patients: a meta-analysis. Frontiers in Neurology 2023; 14. Review. DOI: 10.3389/fneur.2023.1288377.  152. H DL, Bridget L, S HR, et al. Evaluation of the neutrophil-to-lymphocyte and platelet-to-lymphocyte ratios in critically ill dogs. Journal of veterinary emergency and critical care (San Antonio, Tex : 2001) 2022; 33: 52-58.  153. Han Q, Li M, Su D, et al. Development and validation of a 30-day death nomogram in patients with spontaneous cerebral hemorrhage: a retrospective cohort study. Acta Neurologica Belgica 2022; 122: 67-74. Article. DOI: 10.1007/s13760-021-01617-1.  154. Han W, Yi HJ, Shin DS, et al. Pan-immune-inflammation value predict delayed cerebral ischemia in patients with aneurysmal subarachnoid hemorrhage. Journal of Clinical Neuroscience 2024; 121: 47-52. Article. DOI: 10.1016/j.jocn.2024.02.003.  155. Hathidara MY, Campos Y, Chandrashekhar S, et al. Scoring System to Predict Hospital Outcome After Subarachnoid Hemorrhage-Incorporating Systemic Response: The CRIG Score. J Stroke Cerebrovasc Dis 2022; 31: 106577. 20220524. DOI: 10.1016/j.jstrokecerebrovasdis.2022.106577.  156. Hermawan Saputro A, Gofir A, Paryono, et al. P-OT017. Neutrophil-to-lymphocyte ratio, monocyte-to- lymphocyte ratio, and platelet-tolymphocyte ratio biomarkers in severe hemorrhagic stroke patients: Which ratio to choose as a mortality predictor? Clinical Neurophysiology 2021; 132: e126. Conference Abstract. DOI: 10.1016/j.clinph.2021.02.314.  157. Hong SI, Kim JS, Bae HJ, et al. C-reactive Protein for Stroke Detection in the Emergency Department in Patients With Dizziness Without Neurological Deficits. Frontiers in Neurology 2021; 12. Article. DOI: 10.3389/fneur.2021.662510.  158. Huang KH, Chen FY, Liu ZZ, et al. Prediction of pre-eclampsia complicated by fetal growth restriction and its perinatal outcome based on an artificial neural network model. Frontiers in Physiology 2022; 13. Article. DOI: 10.3389/fphys.2022.992040.  159. Hui OS, Yilong Z, Vincent N, et al. 707 Associations Between Inflammatory Markers and Outcomes in SICH. Neurosurgery 2024; 70: 163-163.  160. Huijun W, Ning W, Min L, et al. The early predictive value of platelet-to-lymphocyte ratio to hemorrhagic transformation of young acute ischemic stroke. Asian Biomedicine 2023; 17: 267-272.  161. Ignacio KHD, Diestro JDB, Enriquez CAG, et al. Predictive Value of Hematologic Inflammatory Markers in Delayed Cerebral Ischemia After Aneurysmal Subarachnoid Hemorrhage. World Neurosurgery 2022; 160: e296-e306. Article. DOI: 10.1016/j.wneu.2022.01.014.  162. Inanc Y and Inanc Y. The effects of neutrophil to lymphocyte and platelet to lymphocyte ratios on prognosis in patients undergoing mechanical thrombectomy for acute ischemic stroke. Ann Ital Chir 2018; 89: 367-373.  163. Jabbarli R, Pierscianek D, Darkwah Oppong M, et al. Laboratory biomarkers of delayed cerebral ischemia after subarachnoid hemorrhage: a systematic review. Neurosurgical Review 2020; 43: 825-833. Review. DOI: 10.1007/s10143-018-1037-y.  164. Jaffar DW and Feissal Rabie MA. Maternal platelet-to-lymphocyte ratio at delivery can predict poor neonatal outcome in preterm births. Turkish Journal of Obstetrics and Gynecology 2018; 15: 254-258. Article. DOI: 10.4274/TJOD.65299.  165. Jin H, Peng Q, Li M, et al. Supra-Blan2t score as a multisystem-based risk score to predict poor 3-month outcome in acute ischemic stroke patients with intravenous thrombolysis. CNS Neuroscience and Therapeutics 2024; 30. Article. DOI: 10.1111/cns.14381.  166. Jin M, Wenting G, Jiali X, et al. Association of platelet-to-lymphocyte ratio and neutrophil-to-lymphocyte ratio with outcomes in stroke patients achieving successful recanalization by endovascular thrombectomy&#13. Frontiers in Neurology 2022.  167. Kadiroglu AK, Erginer UM, Gumus MT, et al. Evaluation of effect of the inflammatory biomarkers on mortality in patients with acute stroke in intensive care unit. Journal of Critical Care 2024; 81. Conference Abstract. DOI: 10.1016/j.jcrc.2024.154631.  168. Kalafat UM, Bildik B, Dorter M, et al. Value of neutrophile/lymphocyte ratio for differentiation of stroke cases in the emergency department. Acta Medica Mediterranea 2020; 36: 977-981. Article. DOI: 10.19193/0393-6384_2020_2_154.  169. Karahan SZ, Gazioglu S, Dilaver I, et al. The role of thrombo-inflammatory biomarkers in the prognosis of cerebral venous sinus thrombosis. Current Neurovascular Research 2021; 18: 237-243. Article. DOI: 10.2174/1567202618666210607151518.  170. Kayalar AE, Ozlu EBK, Etli MU, et al. Evaluation of Peripheral Blood Inflammatory Markers in Patients with Chronic Subdural Hematoma. Neurology India 2024; 72: 340-344. Article. DOI: 10.4103/ni.ni_390_22.  171. Kim JH, Yi HJ, Kim BT, et al. Clinical relevance of serum procalcitonin in patients with aneurysmal subarachnoid hemorrhage. Experimental and Therapeutic Medicine 2022; 24. Article. DOI: 10.3892/etm.2022.11590.  172. Kimura T. Neutrophil-to-lymphocyte ratio: Will it improve outcome prediction after stroke? Minerva Anestesiologica 2020; 86: 901-903. Editorial. DOI: 10.23736/S0375-9393.20.14816-8.  173. Kömürcü HF, Gözke E, Salt I, et al. Time‑dependent changes in blood cells, NIHSS and mRS according to reperfusion treatment type in stroke patients who developed hemorrhagic complication. Acta Neurobiol Exp (Wars) 2024; 84: 70-79. 20240328. DOI: 10.55782/ane-2024-2540.  174. Kotfis K, Witkiewicz W, Szylińska A, et al. Delirium severely worsens outcome in patients with covid-19—a retrospective cohort study from temporary critical care hospitals. Journal of Clinical Medicine 2021; 10. Article. DOI: 10.3390/jcm10132974.  175. Kusuma GFP, Maliawan S, Mahadewa TGB, et al. Neutrophil-to-lymphocyte ratio and platelet-to-lymphocyte ratio as an inflammatory biomarker in predicting the severity of secondary brain injury: A review article. Open Access Macedonian Journal of Medical Sciences 2020; 8: 272-282. Review. DOI: 10.3889/oamjms.2020.4789.  176. Kwon S, Jin C, Cho SY, et al. Analysis of Factors Affecting Post-Stroke Fatigue: An Observational, Cross-Sectional, Retrospective Chart Review Study. Healthcare (Basel) 2021; 9 20211119. DOI: 10.3390/healthcare9111586.  177. Lan F, Liu T, Guan C, et al. Nomogram for Risk of Secondary Venous Thromboembolism in Stroke Patients: A Study Based on the MIMIC-IV Database. Clinical and Applied Thrombosis/Hemostasis 2024; 30. Article. DOI: 10.1177/10760296241254104.  178. Li C, Shaohuai X, Yi Z, et al. Systemic immune inflammation index and peripheral blood carbon dioxide concentration at admission predict poor prognosis in patients with severe traumatic brain injury&#13. Frontiers in Immunology 2023; 13: 1034916-1034916.  179. Li F, Chen A, Li Z, et al. Machine learning-based prediction of cerebral hemorrhage in patients with hemodialysis: A multicenter, retrospective study. Frontiers in Neurology 2023; 14. Article. DOI: 10.3389/fneur.2023.1139096.  180. Li R, Lin F, Chen Y, et al. A 90-Day Prognostic Model Based on the Early Brain Injury Indicators after Aneurysmal Subarachnoid Hemorrhage: the TAPS Score. Translational Stroke Research 2023; 14: 200-210. Article. DOI: 10.1007/s12975-022-01033-4.  181. Li Y, Wen D, Cui W, et al. The Prognostic Value of the Acute Phase Systemic Immune-Inflammation Index in Patients With Intracerebral Hemorrhage. Front Neurol 2021; 12: 628557. 20210525. DOI: 10.3389/fneur.2021.628557.  182. Liao B, Xu Q, Lu P, et al. The prognostic value of systemic immune-inflammation index in patients with aneurysmal subarachnoid hemorrhage: a systematic review. Neurosurgical Review 2023; 46. Review. DOI: 10.1007/s10143-023-02133-x.  183. Liu C, Li F, Liu S, et al. Neutrophil Count Predicts Malignant Cerebellar Edema and Poor Outcome in Acute Basilar Artery Occlusion Receiving Endovascular Treatment: A Nationwide Registry-Based Study. Frontiers in Immunology 2022; 13. Article. DOI: 10.3389/fimmu.2022.835915.  184. Liu J, Wang Y, Jin Y, et al. Prediction of Hemorrhagic Transformation After Ischemic Stroke: Development and Validation Study of a Novel Multi-biomarker Model. Front Aging Neurosci 2021; 13: 667934. 20210528. DOI: 10.3389/fnagi.2021.667934.  185. Liu YL, Lu JK, Yin HP, et al. High Neutrophil-to-Lymphocyte Ratio Predicts Hemorrhagic Transformation in Acute Ischemic Stroke Patients Treated with Intravenous Thrombolysis. Int J Hypertens 2020; 2020: 5980261. 20200227. DOI: 10.1155/2020/5980261.  186. Liu Z, Yang C, Wang X, et al. Blood-Based Biomarkers: A Forgotten Friend of Hyperacute Ischemic Stroke. Frontiers in Neurology 2021; 12. Review. DOI: 10.3389/fneur.2021.634717.  187. Lu W, Tong Y, Zhang C, et al. A novel visual dynamic nomogram to online predict the risk of unfavorable outcome in elderly aSAH patients after endovascular coiling: A retrospective study. Frontiers in Neuroscience 2023; 16. Article. DOI: 10.3389/fnins.2022.1037895.  188. Lukito PP, July J, Suntoro VA, et al. Neutrophil-to-lymphocyte ratio predicted cerebral infarction and poor discharge functional outcome in aneurysmal subarachnoid hemorrhage: A propensity score matching analysis. Surgical Neurology International 2023; 14. Article. DOI: 10.25259/SNI_127_2023.  189. Luo F, Li Y, Zhao Y, et al. Systemic immune-inflammation index predicts the outcome after aneurysmal subarachnoid hemorrhage. Neurosurgical Review 2022; 45: 1607-1615. Article. DOI: 10.1007/s10143-021-01681-4.  190. Luo Y and Zhao J. The dynamic changes of peripheral blood cell counts predict the clinical outcomes of aneurysmal subarachnoid hemorrhage. Heliyon 2024; 10: e29763. 20240416. DOI: 10.1016/j.heliyon.2024.e29763.  191. Ma J, Guo W, Xu J, et al. Association of platelet-to-lymphocyte ratio and neutrophil-to-lymphocyte ratio with outcomes in stroke patients achieving successful recanalization by endovascular thrombectomy. Frontiers in Neurology 2022; 13. Article. DOI: 10.3389/fneur.2022.1039060.  192. Magoon R. Routine labs for the prediction of hematoma expansion after intracerebral hemorrhage. American Journal of Emergency Medicine 2023; 72: 213. Letter. DOI: 10.1016/j.ajem.2023.07.054.  193. Matsushita FY, Krebs VLJ and de Carvalho WB. Identifying two distinct subphenotypes of patent ductus arteriosus in preterm infants using machine learning. European Journal of Pediatrics 2023; 182: 2173-2179. Article. DOI: 10.1007/s00431-023-04882-9.  194. Mehmet A, Bülent K, Kerem K, et al. Intraoperative hemorrhage and increased spleen volume are risk factors for conversion to open surgery in patients undergoing elective robotic and laparoscopic splenectomy. Turkish journal of surgery 2020; 36: 72-81.  195. Meshaal MS, Nagi A, Eldamaty A, et al. Neutrophil-to-lymphocyte ratio (NLR) and platelet-to-lymphocyte ratio (PLR) as independent predictors of outcome in infective endocarditis (IE). Egyptian Heart Journal 2019; 71. Article. DOI: 10.1186/s43044-019-0014-2.  196. Min Y, Zhilong X, Huangyan Z, et al. Association between platelet-lymphocyte ratio and 90-day mortality in patients with intracerebral hemorrhage: data from the MIMIC-III database&#13. Frontiers in Neurology 2023; 14: 1234252-1234252.  197. Morga R, Dziedzic T, Moskala M, et al. Clinical Relevance of Changes in Peripheral Blood Cells After Intracranial Aneurysm Rupture. Journal of Stroke and Cerebrovascular Diseases 2020; 29. Article. DOI: 10.1016/j.jstrokecerebrovasdis.2020.105293.  198. Morotti A, Phuah CL, Anderson CD, et al. Leukocyte Count and Intracerebral Hemorrhage Expansion. Stroke 2016; 47: 1473-1478. 20160421. DOI: 10.1161/strokeaha.116.013176.  199. Mustafa O, Bulent B, Ilker KM, et al. Analysis of biochemical laboratory values to determine etiology and prognosis in patients with subarachnoid hemorrhage: a clinical study. Neurological research 2018; 41: 1-12.  200. Nie Z, Lin F, Li R, et al. A Pooled Analysis of Preoperative Inflammatory Biomarkers to Predict 90-Day Outcomes in Patients with an Aneurysmal Subarachnoid Hemorrhage: A Single-Center Retrospective Study. Brain Sciences 2023; 13. Article. DOI: 10.3390/brainsci13020257.  201. Ogden M, Bakar B, Karagedik MI, et al. Analysis of biochemical laboratory values to determine etiology and prognosis in patients with subarachnoid hemorrhage: a clinical study. Neurological Research 2019; 41: 156-167. Article. DOI: 10.1080/01616412.2018.1545414.  202. Parekh A, Satish S, Dulhanty L, et al. Clinical prediction models for aneurysmal subarachnoid haemorrhage: a systematic review update. European Stroke Journal 2023; 8: 384. Conference Abstract. DOI: 10.1177/23969873231169660.  203. Pereira JPdC, Diniz AdS, Lemos MCCd, et al. Prognostic value of the geriatric nutritional risk index and other hematological markers on long-term survival in the geriatric population. Geriatrics & gerontology international 2024; 24: 312-318.  204. Pereira M, Batista R, Marreiros A, et al. Neutrophil-to-leukocyte ratio and admission glycemia as predictors of short-term death in very old elderlies with lobar intracerebral hemorrhage. Brain Circ 2023; 9: 94-98. 20230630. DOI: 10.4103/bc.bc_5_23.  205. Prabhu S and Harshavardhan L. Role of Platelet to Lymphocyte Ratio, Neutrophil to Lymphocyte Ratio and Lymphocyte to Monocyte Ratio in Acute Ischaemic Stroke Severity: A Prospective Cohort Study. Journal of Clinical and Diagnostic Research 2023; 17: 12-16. Article. DOI: 10.7860/JCDR/2023/64152.18614.  206. Qin Y, Liu L, Zhao S, et al. Blood inflammatory biomarkers predict in-hospital pneumonia after endovascular treatment of aneurysm in patients with aneurysmal subarachoid hemorrhage. Neurosurgical Review 2023; 46. Article. DOI: 10.1007/s10143-023-02082-5.  207. Qin Y, Zhang B, Zhao S, et al. Association between higher systemic immune inflammation index (SII) and deep vein thrombosis (DVT) in patients with aneurysmal subarachnoid hemorrhage (aSAH) after endovascular treatment. Neurosurgical Review 2023; 46. Article. DOI: 10.1007/s10143-023-02048-7.  208. Rodrigues M, Costa R, Brito S, et al. Ischemic stroke and cancer correlation: A stroke unit experience. Annals of Oncology 2017; 28: v40. Conference Abstract. DOI: 10.1093/annonc/mdx363.057.  209. Rogers LR. Cerebrovascular complications in cancer patients. Neurol Clin 2003; 21: 167-192. DOI: 10.1016/s0733-8619(02)00066-x.  210. Rzepliński R, Kostyra K, Skadorwa T, et al. Acute platelet response to aneurysmal subarachnoid hemorrhage depends on severity and distribution of bleeding: an observational cohort study. Neurosurgical Review 2021; 44: 2647-2658. Article. DOI: 10.1007/s10143-020-01444-7.  211. Saki N, Javan M, Shokouhian M, et al. A review of the interaction between the coagulation system and cancer. Iranian Journal of Blood and Cancer 2023; 15: 71-79. Review.  212. Salas-Perdomo A, Miró-Mur F, Gallizioli M, et al. Role of the S1P pathway and inhibition by fingolimod in preventing hemorrhagic transformation after stroke. Sci Rep 2019; 9: 8309. 20190605. DOI: 10.1038/s41598-019-44845-5.  213. Salas-Perdomo A, Miró-Mur F, Urra X, et al. T Cells Prevent Hemorrhagic Transformation in Ischemic Stroke by P-Selectin Binding. Arterioscler Thromb Vasc Biol 2018; 38: 1761-1771. DOI: 10.1161/atvbaha.118.311284.  214. Sarioglu O, Capar AE, Bas Sokmez DF, et al. Relationship between the first pass effect and the platelet-lymphocyte ratio in acute ischemic stroke. Interventional Neuroradiology 2021; 27: 523-530. Article. DOI: 10.1177/1591019920976251.  215. Sharma D and Bhaskar SMM. Prognostic Role of the Platelet-Lymphocyte Ratio in Acute Ischemic Stroke Patients Undergoing Reperfusion Therapy: A Meta-Analysis. Journal of Central Nervous System Disease 2022; 14. Article. DOI: 10.1177/11795735221110373.  216. Shimizu Y, Uchiyama S, Mori G, et al. [A young patient with endometrioid adenocarcinoma who suffered Trousseau's syndrome associated with vasculitis]. Rinsho Shinkeigaku 2002; 42: 227-232.  217. Shin KW, Choi S, Oh H, et al. A High Immediate Postoperative Neutrophil-to-Albumin Ratio is Associated With Unfavorable Clinical Outcomes at Hospital Discharge in Patients With Aneurysmal Subarachnoid Hemorrhage. Journal of Neurosurgical Anesthesiology 2024; 36: 142-149. Article. DOI: 10.1097/ANA.0000000000000906.  218. Siempis T, Georgalis PA, Lianos G, et al. Blood Biomarkers for Prediction of Positive CT Findings in Mild Traumatic Brain Injury in Paediatric Population. Journal of Integrative Neuroscience 2023; 22. Article. DOI: 10.31083/j.jin2204091.  219. Skiba I, Kopanitsa G, Metsker O, et al. Application of Machine Learning Methods for Epilepsy Risk Ranking in Patients with Hematopoietic Malignancies Using. J Pers Med 2022; 12 20220811. DOI: 10.3390/jpm12081306.  220. Tian M, Li Y, Wang X, et al. The Hemoglobin, Albumin, Lymphocyte, and Platelet (HALP) Score Is Associated With Poor Outcome of Acute Ischemic Stroke. Front Neurol 2020; 11: 610318. 20210112. DOI: 10.3389/fneur.2020.610318.  221. Tian T, Wang L, Xu J, et al. Prediction of early neurological deterioration in acute ischemic stroke patients treated with intravenous thrombolysis. Journal of Cerebral Blood Flow and Metabolism 2023; 43: 2049-2059. Article. DOI: 10.1177/0271678X231200117.  222. Topcuoglu MA, Pektezel MY, Oge DD, et al. Stroke Mechanism in COVID-19 Infection: A Prospective Case-Control Study. J Stroke Cerebrovasc Dis 2021; 30: 105919. 20210601. DOI: 10.1016/j.jstrokecerebrovasdis.2021.105919.  223. Topcuoglu MA, Pektezel MY, Yilmaz E, et al. Systemic Inflammation Indices in Patients With Acute Ischemic Stroke Treated With Intravenous Tissue Plasminogen Activator: Clinical Yield and Utility. Angiology 2021; 72: 279-284. Article. DOI: 10.1177/0003319720969997.  224. Trifan G and Testai FD. Systemic Immune-Inflammation (SII) index predicts poor outcome after spontaneous supratentorial intracerebral hemorrhage. J Stroke Cerebrovasc Dis 2020; 29: 105057. 20200626. DOI: 10.1016/j.jstrokecerebrovasdis.2020.105057.  225. Uzunkaya F and Idil Soylu A. Predictors of full functional recovery in endovascularly treated patients with aneurysmal subarachnoid hemorrhage. Turkish Journal of Medical Sciences 2021; 51: 2000-2006. Article. DOI: 10.3906/sag-2103-3.  226. Vardar G and Ozek E. Diagnostic utility of the systemic immune-inflammatory index in preterm neonates with late-onset sepsis. Marmara Medical Journal 2023; 36: 319-325. Article. DOI: 10.5472/marumj.1367904.  227. Vidale S, Bellocchi S and Arnaboldi M. Platelet and neutrophil iymphocyte ratios at admission and the clinical outcome at discharge in haemorrhagic strokes. Cerebrovascular Diseases 2017; 43: 80. Conference Abstract.  228. Waeeb JD and Abubakr FRM. Maternal platelet-to-lymphocyte ratio at delivery can predict poor neonatal outcome in preterm births. Turkish journal of obstetrics and gynecology 2018; 15: 254-258.  229. Wang K, Li R, Chen X, et al. Platelet-to-white blood cell ratio: A feasible predictor for unfavorable functional outcome in patients with aneurysmal subarachnoid hemorrhage. Journal of Clinical Neuroscience 2023; 115: 108-113. Article. DOI: 10.1016/j.jocn.2023.07.019.  230. Wang R, He M, Ou X, et al. CRP Albumin ratio is positively associated with poor outcome in patients with traumatic brain injury. Clinical Neurology and Neurosurgery 2020; 195. Article. DOI: 10.1016/j.clineuro.2020.106051.  231. Wang X, Zhao Y, Zhao J, et al. Neutrophil–lymphocyte ratio on first and third postoperative days: associated with severe pneumonia in aneurysmal subarachnoid hemorrhage patients undergoing surgeries. Neurosurgical Review 2024; 47. Article. DOI: 10.1007/s10143-024-02295-2.  232. Wang X, Zhao Y, Zhao J, et al. Neutrophil-lymphocyte ratio on first and third postoperative days: associated with severe pneumonia in aneurysmal subarachnoid hemorrhage patients undergoing surgeries. Neurosurgical review 2024; 47: 70-70.  233. Wei F and Qin W. Comparison of statistical analysis using the random-effects and inverse variance heterogeneity models for a meta-analysis. International immunopharmacology 2022; 108: 108884-108884.  234. Wu CR, Chen JS, Chen YS, et al. Nomogram for the Prediction of Shunt-Dependent Hydrocephalus in Patients with Aneurysmal Subarachnoid Hemorrhage: A Single-institute Experience. 2023.  235. Wu D, Sha Z, Fan Y, et al. Evaluating the efficiency of a nomogram based on the data of neurosurgical intensive care unit patients to predict pulmonary infection of multidrug-resistant Acinetobacter baumannii. Frontiers in Cellular and Infection Microbiology 2023; 13. Article. DOI: 10.3389/fcimb.2023.1152512.  236. Xiao H, Li L, Zhang F, et al. Preoperative systemic immune-inflammation index may predict prolonged mechanical ventilation in patients with spontaneous basal ganglia intracerebral hemorrhage undergoing surgical operation. Front Neurol 2023; 14: 1190544. 20230615. DOI: 10.3389/fneur.2023.1190544.  237. Xu J, Guo W, Ma J, et al. Preceding transient ischemic attack was associated with functional outcome after stroke thrombectomy: A propensity score matching study. Journal of Cerebral Blood Flow and Metabolism 2023; 43: 1390-1399. Article. DOI: 10.1177/0271678X231167924.  238. Xu M, Chen R, Liu L, et al. Systemic immune-inflammation index and incident cardiovascular diseases among middle-aged and elderly Chinese adults: The Dongfeng-Tongji cohort study. Atherosclerosis 2021; 323: 20-29. 20210220. DOI: 10.1016/j.atherosclerosis.2021.02.012.  239. Yamakawa K, Umemura Y, Murao S, et al. Optimal Timing and Early Intervention With Anticoagulant Therapy for Sepsis-Induced Disseminated Intravascular Coagulation. Clinical and Applied Thrombosis/Hemostasis 2019; 25. Article. DOI: 10.1177/1076029619835055.  240. Yang X, Wang K, Shen P, et al. Association of plasma sphingosine-1-phosphate levels with disease severity and prognosis after intracerebral hemorrhage. Frontiers in Neurology 2024; 15. Article. DOI: 10.3389/fneur.2024.1365902.  241. Yang Y, Xie D and Zhang Y. Increased Platelet-to-Lymphocyte Ratio is an Independent Predictor of Hemorrhagic Transformation and In-Hospital Mortality Among Acute Ischemic Stroke with Large-Artery Atherosclerosis Patients. Int J Gen Med 2021; 14: 7545-7555. 20211101. DOI: 10.2147/ijgm.S329398.  242. Ye Z, Hu T, Wang J, et al. Systemic immune-inflammation index as a potential biomarker of cardiovascular diseases: A systematic review and meta-analysis. Frontiers in Cardiovascular Medicine 2022; 9. Review. DOI: 10.3389/fcvm.2022.933913.  243. Yi Y, Dan X and Yongbo Z. Increased Platelet-to-Lymphocyte Ratio is an Independent Predictor of Hemorrhagic Transformation and In-Hospital Mortality Among Acute Ischemic Stroke with Large-Artery Atherosclerosis Patients. International journal of general medicine 2021; 14: 7545-7555.  244. Yongfeng Z, Xian W, Hongbo R, et al. Systemic inflammation response index (SIRI) on the 3rd postoperative day are associated with severe pneumonia in cerebral hemorrhage patients: A single-center retrospective study. Medicine 2023; 102: e35587-.  245. Yongkai Q, Baorui Z, Shangfeng Z, et al. Association between higher systemic immune inflammation index (SII) and deep vein thrombosis (DVT) in patients with aneurysmal subarachnoid hemorrhage (aSAH) after endovascular treatment. Neurosurgical Review 2023; 46: 142-142.  246. Yongkai Q, Lang L, Shangfeng Z, et al. Blood inflammatory biomarkers predict in-hospital pneumonia after endovascular treatment of aneurysm in patients with aneurysmal subarachoid hemorrhage. Neurosurgical review 2023; 46: 171-171.  247. Yu T, Liu H, Liu Y, et al. Inflammatory response biomarkers nomogram for predicting pneumonia in patients with spontaneous intracerebral hemorrhage. Front Neurol 2022; 13: 1084616. 20230112. DOI: 10.3389/fneur.2022.1084616.  248. Yu T, Liu H, Liu Y, et al. Inflammatory response biomarkers nomogram for predicting pneumonia in patients with spontaneous intracerebral hemorrhage. Frontiers in Neurology 2023; 13. Article. DOI: 10.3389/fneur.2022.1084616.  249. Yun S, Yi HJ, Lee DH, et al. Systemic Inflammation Response Index and Systemic Immune-inflammation Index for Predicting the Prognosis of Patients with Aneurysmal Subarachnoid Hemorrhage. Journal of Stroke and Cerebrovascular Diseases 2021; 30. Article. DOI: 10.1016/j.jstrokecerebrovasdis.2021.105861.  250. Yusuf I and Yılmaz I. The effects of neutrophil to lymphocyte and platelet to lymphocyte ratios on prognosis in patients undergoing mechanical thrombectomy for acute ischemic stroke. Annali italiani di chirurgia 2018; 89: 367-373.  251. Zawiah M, Khan AH, Abu Farha R, et al. Predictors of stroke-associated pneumonia and the predictive value of neutrophil percentage-to-albumin ratio. Postgraduate Medicine 2023; 135: 681-689. Article. DOI: 10.1080/00325481.2023.2261354.  252. Zhang P, Li Y, Zhang H, et al. Prognostic value of the systemic inflammation response index in patients with aneurismal subarachnoid hemorrhage and a Nomogram model construction. British Journal of Neurosurgery 2023; 37: 1560-1566. Article. DOI: 10.1080/02688697.2020.1831438.  253. Zhang Q, Zhang G, Wang L, et al. Clinical Value and Prognosis of C Reactive Protein to Lymphocyte Ratio in Severe Aneurysmal Subarachnoid Hemorrhage. Frontiers in Neurology 2022; 13. Review. DOI: 10.3389/fneur.2022.868764.  254. Zhao Y, Wang X, Ren H, et al. Systemic inflammation response index (SIRI) on the 3rd postoperative day are associated with severe pneumonia in cerebral hemorrhage patients: A single-center retrospective study. Medicine (United States) 2023; 102: E35587. Article. DOI: 10.1097/MD.0000000000035587.  255. Zhu Y, Xie Z, Shen J, et al. Association between systemic inflammatory response syndrome and hematoma expansion in intracerebral hemorrhage. Advances in Clinical and Experimental Medicine 2022; 31. Article. DOI: 10.17219/ACEM/145852.  256. Zhuang D, Ren Z, Sheng J, et al. A dynamic nomogram for predicting unfavorable prognosis after aneurysmal subarachnoid hemorrhage. Annals of Clinical and Translational Neurology 2023; 10: 1058-1071. Article. DOI: 10.1002/acn3.51789. |
| **Reports not retrieved (n=8)** |
| 1. 房鸿彬. *中性粒细胞-淋巴细胞比值等炎症标志物与高血压脑出血患者预后的相关性研究*. 硕士, 2021.  2. 揭伟, 刘微波, 刘莎, et al. 蛛网膜下腔出血患者外周血NLR、LMR、PLR和SII值变化的临床意义. *脑与神经疾病杂志* 2024; 32: 345-349.  3. 陶冶, 薛维爽 and 滕伟禹. 脑出血患者外周血中性粒细胞/淋巴细胞和血小板/淋巴细胞比值的临床意义. *中国现代医学杂志* 2017; 27: 80-84.  4. 姚师. *NLR值和PLR值与动脉瘤性蛛网膜下腔出血患者关系的探讨*. 硕士, 2022.  5. Yilmaz A and Özkul A. Admission neutrophil to lymphocyte and platelet to lymphocyte ratio as a predictor of mortality in patients with subarachnoid hemorrhage. *Turk Beyin Damar Hastaliklar Dergisi* 2018; 24: 19-25. Article. DOI: 10.5505/tbdhd.2018.57338.  6. Hermawan Saputro A, Gofir A, Paryono, et al. P-OT017. Neutrophil-to-lymphocyte ratio, monocyte-to- lymphocyte ratio, and platelet-tolymphocyte ratio biomarkers in severe hemorrhagic stroke patients: Which ratio to choose as a mortality predictor? *Clinical Neurophysiology* 2021; 132: e126. Conference Abstract. DOI: 10.1016/j.clinph.2021.02.314.  7. 庞博 and 林兴栋. 自发性脑出血患者的血小板/淋巴细胞比及单核细胞/淋巴细胞比值的临床意义. In: *世界中医药学会联合会老年医学专业委员会、中国中西医结合学会慢病防治与管理专业委员会2019学术年会* 中国北京, 2019, p.1.  8. 庞琪, 杨合慧, 曾怀文, et al. 血液指标在自发性脑出血中的临床意义. *江苏医药* 2024; 50: 245-249. DOI: 10.19460/j.cnki.0253-3685.2024.03.007. |
| **Without survival information (n=11)** |
| 1. Bacigaluppi S, Bragazzi NL, Ivaldi F, et al. Systemic Inflammatory Response in Spontaneous Subarachnoid Hemorrhage from Aneurysmal Rupture versus Subarachnoid Hemorrhage of Unknown Origin. *Journal of Inflammation Research* 2022; 15: 6329-6342. Article. DOI: 10.2147/JIR.S380101.  2. Bolton WS, Gharial PK, Akhunbay-Fudge C, et al. Day 2 neutrophil-to-lymphocyte and platelet-to-lymphocyte ratios for prediction of delayed cerebral ischemia in subarachnoid hemorrhage. *Neurosurgical focus* 2022; 52: E4. Article. DOI: 10.3171/2021.12.FOCUS21642.  3. de OAJM, Nunes RN, Mota TJP, et al. Neutrophil-to-lymphocyte and platelet-to-lymphocyte ratios and prognosis after aneurysmal subarachnoid hemorrhage: a cohort study. *Arquivos de neuro-psiquiatria* 2023; 81: 515-523.  4. Department of Neurosurgery TFPsHoWD, Suzhou, Jiangsu 215200, P.R. China., Department of Neurosurgery TFPsHoWD, Suzhou, Jiangsu 215200, P.R. China., Department of Neurosurgery TFPsHoWD, Suzhou, Jiangsu 215200, P.R. China., et al. Clinical significance of neutrophil to lymphocyte ratio and platelet to lymphocyte ratio in acute cerebral hemorrhage with gastrointestinal hemorrhage, and logistic regression analysis of risk factors. *Experimental and therapeutic medicine* 2019; 18: 1533-1538.  5. Hong WR, Xin WW, Ping JZ, et al. The clinical value of neutrophil-to-lymphocyte ratio (NLR), systemic immune-inflammation index (SII), platelet-to-lymphocyte ratio (PLR) and systemic inflammation response index (SIRI) for predicting the occurrence and severity of pneumonia in patients with intracerebral hemorrhage&#13. *Frontiers in Immunology* 2023; 14: 1115031-1115031.  6. Jian Z, Chunlong L, Yaofeng H, et al. The trend of neutrophil-to-lymphocyte ratio and platelet-to-lymphocyte ratio in spontaneous intracerebral hemorrhage and the predictive value of short-term postoperative prognosis in patients. *Frontiers in neurology* 2023; 14: 1189898-1189898.  7. Luo S, Yang WS, Shen YQ, et al. The clinical value of neutrophil-to-lymphocyte ratio, platelet-to-lymphocyte ratio, and D-dimer-to-fibrinogen ratio for predicting pneumonia and poor outcomes in patients with acute intracerebral hemorrhage. *Frontiers in Immunology* 2022; 13. Article. DOI: 10.3389/fimmu.2022.1037255.  8. S BW, Kaur GP, Christopher A, et al. Day 2 neutrophil-to-lymphocyte and platelet-to-lymphocyte ratios for prediction of delayed cerebral ischemia in subarachnoid hemorrhage. *Neurosurgical focus* 2022; 52: E4-E4.  9. Shaafi S, Bonakdari E, Sadeghpour Y, et al. Correlation between red blood cell distribution width, neutrophil to lymphocyte ratio, and neutrophil to platelet ratio with 3-month prognosis of patients with intracerebral hemorrhage: a retrospective study. *BMC Neurol* 2022; 22: 191. 20220524. DOI: 10.1186/s12883-022-02721-2.  10. Vidale S, Bellocchi S and Arnaboldi M. Platelet and neutrophil iymphocyte ratios at admission and the clinical outcome at discharge in haemorrhagic strokes. *Cerebrovascular Diseases* 2017; 43: 80. Conference Abstract.  11. 纪荣庄, 李克乐, 林添辉, et al. 血小板和淋巴细胞比率与高血压脑出血的关系. *南昌大学学报(医学版)* 2017; 57: 45-47. |
| **Insufficient data (n=6)** |
| 1. Yildiz E, Kahveci O, Ulu S, et al. Can neutrophil-lymphocyte, platelet-lymphocyte ratio and mean platelet volume be marker in tonsil hemorrhage? *Medicine Science \| International Medical Journal* 2019: 1.  2. Yuezhan Z, Peng Y, Bin G, et al. Comparison of the Diagnostic Values of Neutrophil, Neutrophil to Lymphocyte Ratio, and Platelet to Lymphocyte Ratio in Distinguishing Spontaneous Subarachnoid Hemorrhage from Nontraumatic Acute Headache. *Clinical laboratory* 2019; 65.  3. Yun S, Yi HJ, Lee DH, et al. Systemic Inflammation Response Index and Systemic Immune-inflammation Index for Predicting the Prognosis of Patients with Aneurysmal Subarachnoid Hemorrhage. *Journal of Stroke and Cerebrovascular Diseases* 2021; 30. Article.  4. Zhang P, Li Y, Zhang H, et al. Prognostic value of the systemic inflammation response index in patients with aneurismal subarachnoid hemorrhage and a Nomogram model construction. *British Journal of Neurosurgery* 2023; 37: 1560-1566. Article.  5. Zhang W and Shen Y. Platelet-to-lymphocyte ratio as a new predictive index of neurological outcomes in patients with acute intracranial hemorrhage: A retrospective study. *Medical Science Monitor* 2018; 24: 4413-4420. Article.  6. Zou YU, Zhang WEI, Huang C, et al. Clinical significance of neutrophil to lymphocyte ratio and platelet to lymphocyte ratio in acute cerebral hemorrhage with gastrointestinal hemorrhage, and logistic regression analysis of risk factors. *Experimental and Therapeutic Medicine* 2019; 18: 1533-1538. Article. |
| **Studies included in review (n=6)** |
| 1. Chu H, Huang C, Zhou Z, et al. Inflammatory score predicts early hematoma expansion and poor outcomes in patients with intracerebral hemorrhage. *International journal of surgery (London, England)* 2023; 109: 266-276. Article. DOI: 10.1097/JS9.0000000000000191.  2. Min Y, Zhilong X, Huangyan Z, et al. Association between platelet-lymphocyte ratio and 90-day mortality in patients with intracerebral hemorrhage: data from the MIMIC-III database&#13. *Frontiers in Neurology* 2023; 14: 1234252-1234252.  3. Seonyong Y, Ho JY, Dong HL, et al. Clinical significance of platelet to neutrophil ratio and platelet to lymphocyte ratio in patients with aneurysmal subarachnoid hemorrhage. *Journal of Clinical Neuroscience* 2021; 92: 49-54.  4. Tao C, Wang J, Hu X, et al. Clinical Value of Neutrophil to Lymphocyte and Platelet to Lymphocyte Ratio After Aneurysmal Subarachnoid Hemorrhage. *Neurocritical Care* 2017; 26: 393-401. Article. DOI: 10.1007/s12028-016-0332-0.  5. Weimin Z and Yanfei S. Platelet-to-Lymphocyte Ratio as a New Predictive Index of Neurological Outcomes in Patients with Acute Intracranial Hemorrhage: A Retrospective Study. *Medical science monitor : international medical journal of experimental and clinical research* 2018; 24: 4413-4420.  6. Yejin K, JongHee S, Chulho K, et al. The Clinical Value of Neutrophil-to-Lymphocyte Ratio and Platelet-to-Lymphocyte Ratio for Predicting Hematoma Expansion and Poor Outcomes in Patients with Acute Intracerebral Hemorrhage. *Journal of clinical medicine* 2023; 12. |
